# Functional Humanization of Mouse Alox15 Does Not Alter Early Vascular Lipid Deposition in ApoE-Deficient Mice

**DOI:** 10.3390/ijms27219480

**Published:** 2026-10-23

**Authors:** Jiaxing Yang, Astrid Borchert, Dilyana Branimirova Mangarova, Zixiao Zhao, Bowen Wang, Hartmut Kühn

**Affiliations:** 1 Department of Biochemistry, Charité—Universitätsmedizin Berlin, Corporate Member of Freie Universität Berlin and Humboldt Universität zu Berlin, Charitéplatz 1, 10117 Berlin, Germany; jiaxing.yang@charite.de (J.Y.); astrid.borchert@charite.de (A.B.); 2 Department of Radiology, Charité—Universitätsmedizin Berlin, Corporate Member of Freie Universität Berlin and Humboldt Universität zu Berlin, Charitéplatz 1, 10117 Berlin, Germany; dilyana.mangarova@charite.de; 3 Clinical Research Center for Hair and Skin Science, Department of Dermatology and Allergy, Charité—Universitätsmedizin Berlin, Corporate Member of Freie Universität Berlin and Humboldt Universität zu Berlin, Charitéplatz 1, 10117 Berlin, Germany; zixiao.zhao@charite.de; 4 Urology Surgery Department, The First Affiliated Hospital of Harbin Medical University, 37 Youzheng Street, Nangang District, Harbin 150001, China; wangbowen@hrbmu.edu.cn

**Keywords:** eicosanoids, lipoxygenase, atherosclerosis, cholesterol metabolism, inflammation, ferroptosis

## Abstract

Arachidonic acid 15-lipoxygenases (ALOX15) have previously been implicated in atherogenesis, and functional inactivation of the *Alox15* gene protected mice from arterial lipid deposition. However, translation of these results into humans was criticized since human and mouse ALOX15 orthologs exhibit distinct catalytic properties. We recently created Alox15 knock-in mice (Alox15^ki/ki^), which express an enzyme variant (Leu353Phe mutant) exhibiting a reaction specificity similar to that of human ALOX15. To make these animals athero-susceptible, we crossed them with ApoE^−/−^ mice yielding Alox15^ki/ki^+ApoE^−/−^ animals and compared the degree of hypercholesterolemia, the extent of arterial lipid deposition, and the expression patterns of inflammation-related genes with the corresponding parameters of age- and sex-matched outbred control animals (Alox15^wt/wt^ + ApoE^−/−^) keeping the mice for different time periods on regular chow diet. Peritoneal macrophages of Alox15^ki/ki^ + ApoE^−/−^ mice oxygenated arachidonic acid mainly to the 15S-hydroperoxy derivative (15S-HpETE) and this data indicates systemic functional humanization of the reaction specificity of the Alox15 pathway. In 14- and 25-week-old animals of either sex, we observed consistent genotype-specific differences neither in the extent of aortic lipid deposition nor in the vascular expression patterns of inflammation-related gene products. This data suggests that the reaction specificity of Alox15 may not be of major relevance for early mouse atherogenesis.

## 1. Introduction

The lipoxygenases (ALOX isoforms) are non-heme iron-containing lipid dioxygenases that catalyze regio- and stereo-specific oxygenation of polyunsaturated fatty acids (PUFAs) [1,2]. The primary ALOX products can be further converted to more complex lipid mediators, which include pro-inflammatory compounds such as leukotrienes [3], eoxins [4], and hepoxilins [5], but also anti-inflammatory and/or pro-resolving mediators (SPMs) [6]. In mammals, ALOX isoforms have been implicated in cell differentiation [7,8] and ferroptosis [9] but also in the pathogenesis of cardiovascular [10], neurological [11], and metabolic diseases [12]. The human genome contains six ALOX genes and each gene encodes a functionally distinct ALOX isoform [13]. The mouse genome contains an orthologous gene for each human ALOX gene, but in addition an Aloxe12 gene has been identified [13]. Although human and mouse ALOX15 orthologs share a high degree of structural (similar molecular weights, similar two-domain structure, >80% of amino acid conservation) and functional similarity (similar affinities for PUFAs and oxygen, similar reaction kinetics, similar activation and inactivation behavior), their reaction specificities with different PUFAs (AA, EPA, DHA) are clearly different [14]. For instance, human ALOX15 oxygenates arachidonic acid (AA) preferentially at carbon 15 of the fatty acid hydrocarbon chain forming 15S-HpETE [15]. In contrast, like most mammalian ALOX15 orthologs [16], mouse Alox15 is an AA 12S-lipoxygenating enzyme converting free and esterified AA mainly to 12S-HpETE [17]. The molecular basis for this functional difference has been explored [18], and Leu353Phe exchange completely humanized the reaction specificity of recombinant mouse Alox15 [19]. Genetically modified mice that express the Leu353Phe mutant of mouse Alox15 instead of the wild-type enzyme (Alox15^ki/ki^ mice) were protected in the dextran sodium sulfate (DSS)-induced colitis model [20].

Atherosclerosis is a chronic inflammatory disease of the arterial wall, in which lipid oxidation and immune activation drive lipid deposition in the arterial wall [21]. ALOX15 has been implicated in atherogenesis [22], but the precise role of the enzyme remains controversial [23]. Epidemiological studies related different polymorphisms in the ALOX15 gene to human atherosclerosis [24,25,26,27]. In mice and rabbits, different loss-of-function and gain-of-function strategies suggested pro- [28,29,30,31,32] or anti-atherogenic activities [33,34] for ALOX15. The confusing picture on the pathophysiological role of mammalian ALOX15 orthologs in atherogenesis might be related to the different functional properties of human and rabbit ALOX15 on one hand and mouse Alox15 on the other. This functional difference may be of biological relevance since the PUFA metabolite patterns formed by AA 12-lipoxygenating and AA 15-lipoxygenating ALOX15 orthologs are different.

To investigate whether functional humanization of mouse Alox15 may alter early vascular lipid deposition and vascular inflammation, we crossed the humanized Alox15^ki/ki^ mice [19] into an ApoE^−/−^ background and quantified the extent of vascular lipid deposition and the expression patterns of inflammation related gene products. We found that functional humanization of Alox15 did not consistently alter either early vascular lipid deposition or the pattern of expression of vascular inflammatory genes.

## 2. Results

### 2.1. Functional Humanization of Mouse Alox15 Improves the Lipoprotein Oxygenase Activity of the Recombinant Enzymes

**Figure 1:**
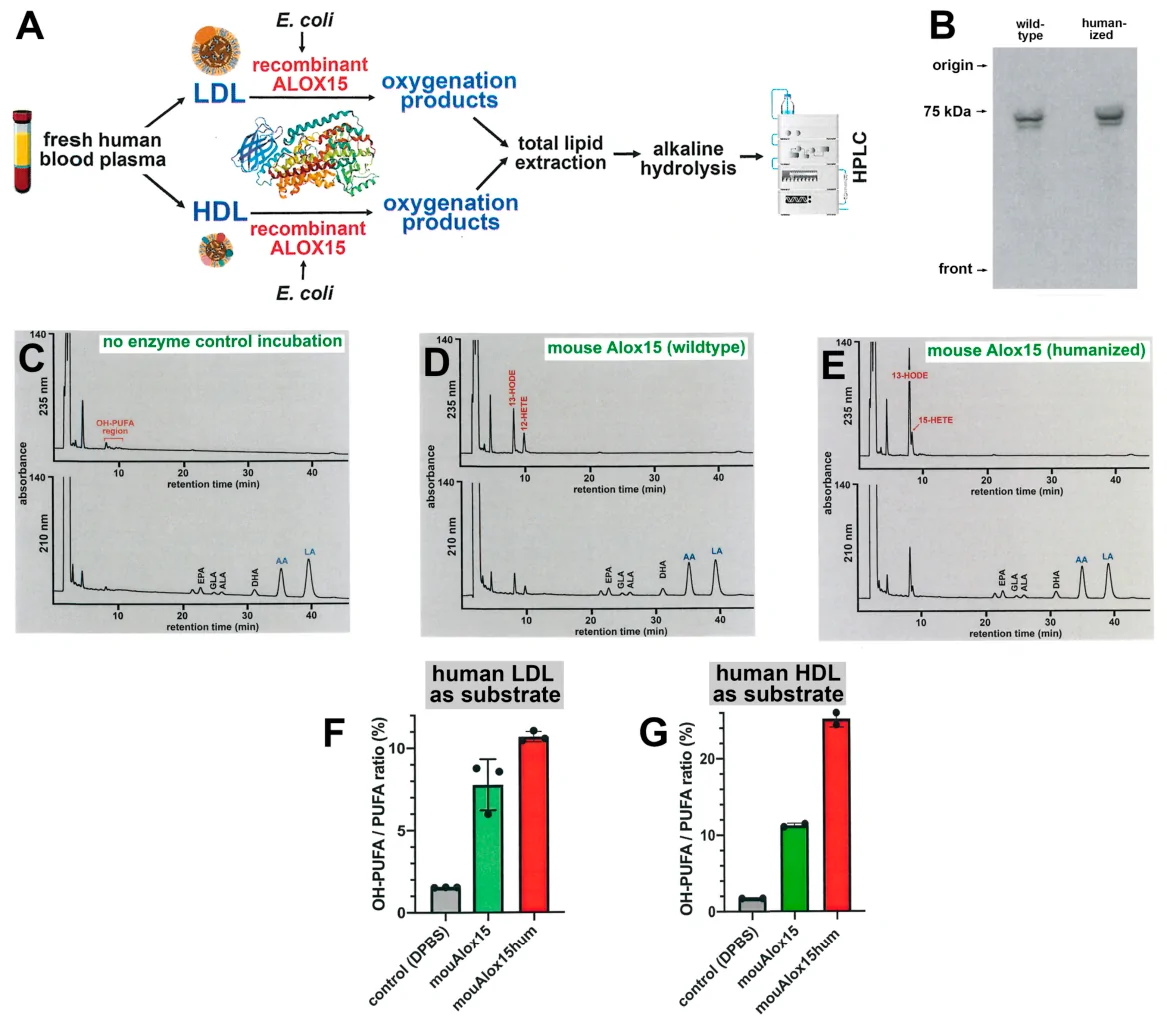
In vitro lipoprotein oxygenase activities of mouse Alox15 variants. In vitro lipoprotein oxygenase activity assays were carried out (see Section 4). For LDL oxygenase activities three activity assays (n = 3) using three independent enzyme preparations were run and representative HPLC chromatograms are shown. For HDL oxygenation two activity assays (n = 2) were carried out using two independent enzyme preparations. (**A**) Experimental strategy (substrate preparation, enzyme preparation, incubation and product formation, sample work-up, RP-HPLC analysis of the oxygenation products). (**B**) Western-blot analysis of recombinant mouse Alox15 orthologs expressed in *E. coli*. (**C**) In vitro LDL oxygenase activity assay; no-enzyme control incubation. Similar chromatograms were obtained when the in vitro lipoprotein oxygenase activity assays were carried out with heated (5 min at 90 °C) aliquots of the bacterial lysate supernatant and with a similar aliquot prepared from competent E. coli cells that were transformed with an empty (non-recombinant) expression plasmid. (**D**) In vitro LDL oxygenase activity; recombinant wild-type mouse Alox15. (**E**) In vitro LDL oxygenase activity; Leu353Phe mutant of mouse Alox15. (**F**) In vitro oxygenase activities of mouse Alox15 variants with human LDL as substrate. (**G**) In vitro oxygenase activities of mouse Alox15 variants with human HDL as substrate. DHA, docosahexaenoic acid; AA, arachidonic acid; LA, linoleic acid; EPA, eicosapentaenoic acid; GLA, gamma-linolenic acid; ALA, alpha-linolenic acid.

Mammalian ALOX15 orthologs have been implicated in atherogenesis, and two alternative mechanistic scenarios have been suggested: (i) Mammalian ALOX15 orthologs are capable of oxygenating native LDL into an atherogenic form [35]. (ii) Mammalian ALOX15 orthologs have been implicated in the formation of pro-resolving eicosanoids [34]. When compared with its human ortholog, mouse Alox15 exhibits a lower biosynthetic capacity for pro-resolving mediators [16,36]. The lipoprotein oxygenase activity of human ALOX15 is higher than that of the mouse ortholog [37], but the impact of functional humanization of mouse Alox15 on this catalytic activity has not been investigated. To address this point, we expressed mouse Alox15 and its humanized Leu353Phe mutant as recombinant proteins, normalized the enzyme preparations to the same amounts of recombinant Alox15, incubated the enzyme preparations with human lipoproteins and analyzed the degree of oxygenation of the lipoprotein lipids (Figure 1A).

In Figure S2 (Supplementary Material), a quantitative immunoblot is shown, and the expression levels of the different enzyme variants were calculated in mg ALOX15 protein per L bacterial liquid culture. Since wild-type mouse Alox15 was somewhat less efficiently expressed than the Leu353Phe mutant (Figure 1B), higher volumes of the bacterial lysate supernatant of the wild-type enzyme preparation were used for the lipoprotein oxygenase activity assay. In no-enzyme control incubations (no bacterial lysate supernatant was added to the activity assay), we only detected small amounts of oxygenated PUFAs (Figure 1C, upper trace), but large amounts of non-oxidized linoleic acid (LA) and arachidonic acid (AA) were observed (Figure 1C, lower trace). In contrast, when recombinant mouse Alox15 was present, large amounts of oxygenated PUFAs (13-HODE, 12-HETE) were present (Figure 1D, upper trace). The pattern of non-oxidized PUFA (Figure 1D, lower trace) was hardly modified. When the Leu353Phe mutant of mouse Alox15 was added (Figure 1E, upper trace), 13-HODE and 15-HETE were identified as the major oxygenation products (no 12-HETE formation) and this data is consistent with the functional humanization of mouse Alox15. Here again, the pattern of non-oxidized PUFA was not altered (Figure 1E, lower trace). To quantitatively compare the LDL oxygenase activities of the two mouse Alox15 variants, we calculated the OH-PUFA/PUFA ratios as a suitable measure for the degree of oxygenation of the LDL lipids (Figure 1F). This data indicates that the LDL oxygenase activity of mouse Alox15 was similar to that of the Leu353Phe mutant. Thus, functional humanization of mouse Alox15 hardly modified the LDL oxygenase activity of the enzyme.

Identical experiments were subsequently performed with isolated human HDL and similar reaction products (13-HODE, 15-HETE, 12-HETE) were identified (Figure S3). With this substrate, wild-type mouse Alox15 was a somewhat less efficient catalyst (Figure 1G). To confirm the ALOX15 origin of the lipoprotein oxygenation products, we quantified their enantiomer compositions and found a high degree of enantioselectivity (Figure S4).

### 2.2. In Vivo Leu353Phe Exchange in the Mouse Alox15 Gene Leads to Functional Humanization of the Enzyme in ApoE-Deficient Background

**Figure 2:**
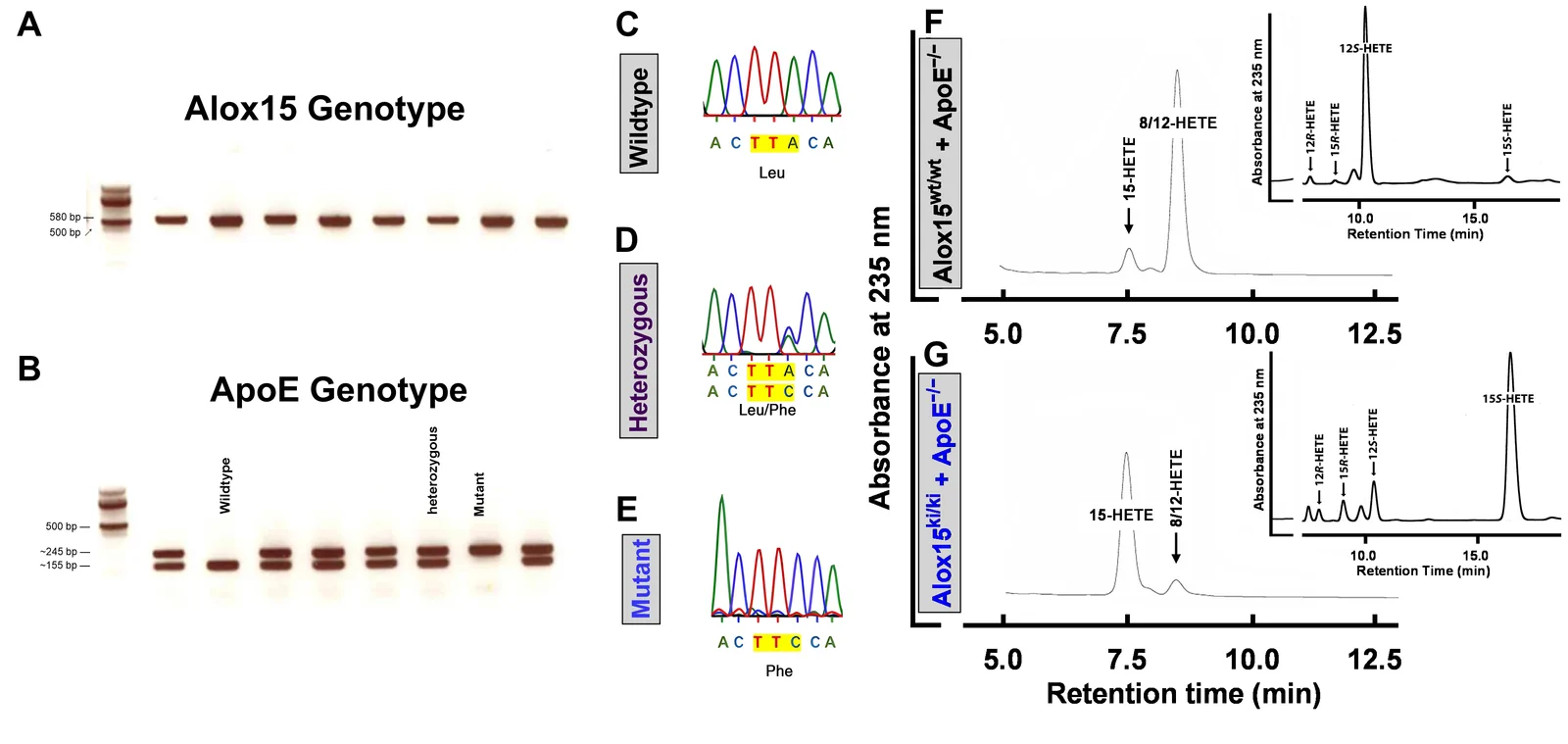
In vivo point mutation in the Alox15 gene leads to functional humanization of mouse Alox15 in ApoE-deficient mice. (**A**) Agarose gel electrophoresis of the PCR products of the Alox15 gene locus. Since the amplification primers did not discriminate between the two genotypes, a single 580 bp product was observed. (**B**) For genotyping of the ApoE gene locus, allele-specific amplification primers were used. The bands of different size identified the wild-type allele (~155 bp) and the knockout allele (~245 bp). (**C**–**E**) Representative sequencing chromatograms of the Alox15 gene locus of Alox15^wt/wt^ (**C**), Alox15wt/ki (**D**), and Alox15ki/ki (**E**) mice. (**F**) Ex vivo Alox15 activity assays using Alox15^wt/wt^+ApoE^−/−^ peritoneal lavage cells. RP-HPLC analysis indicated dominant formation of 12S-HETE (n = 3). Inset: Enantiomer separation of 12-HETE. (**G**) Ex vivo Alox15 activity assays using Alox15^ki/ki^+ApoE^−/−^ peritoneal lavage cells. RP-HPLC analysis indicated dominant formation of 15S-HETE (n = 3). Inset: Enantiomer separation of 15-HETE.

To confirm that our genetic manipulation induced functional humanization of mouse Alox15 in the ApoE-deficient background, we genotyped all individuals included in the study for the two gene loci by genomic PCR. For genotyping of the Alox15 gene locus, the amplification products (Figure 2A) were sequenced (Figure 2C–E) to differentiate between the wild-type and the mutant alleles.

For genotyping of the ApoE locus, we employed allele-specific primer combinations and the size of the amplification products was different for wild-type and knockout alleles (Figure 2B). To confirm functional humanization of mouse Alox15, we prepared peritoneal lavage cells from three different mice of either genotype, incubated the cells with 100 µM AA and analyzed the Alox15 products by RP-HPLC. As expected, peritoneal lavage cells of Alox15^wt/wt^+ApoE^−/−^ mice (Figure 2F) formed 12S-hydroxy-eicosatetraenoic acid (12S-HETE) as the major product. In contrast, 15S-hydroxy-eicosatetraenoic acid (15S-HETE) was the major AA oxygenation product formed by Alox15^ki/ki^+ApoE^−/−^ peritoneal lavage cells (Figure 2G). The total yields of HETE formation were similar for the two genotypes, suggesting similar AA oxygenase activities of wild-type mouse Alox15 and its functionally humanized Leu353Phe mutant.

### 2.3. Functional Humanization of Mouse Alox15 Does Not Induce Major Phenotypic Alterations

**Figure 3:**
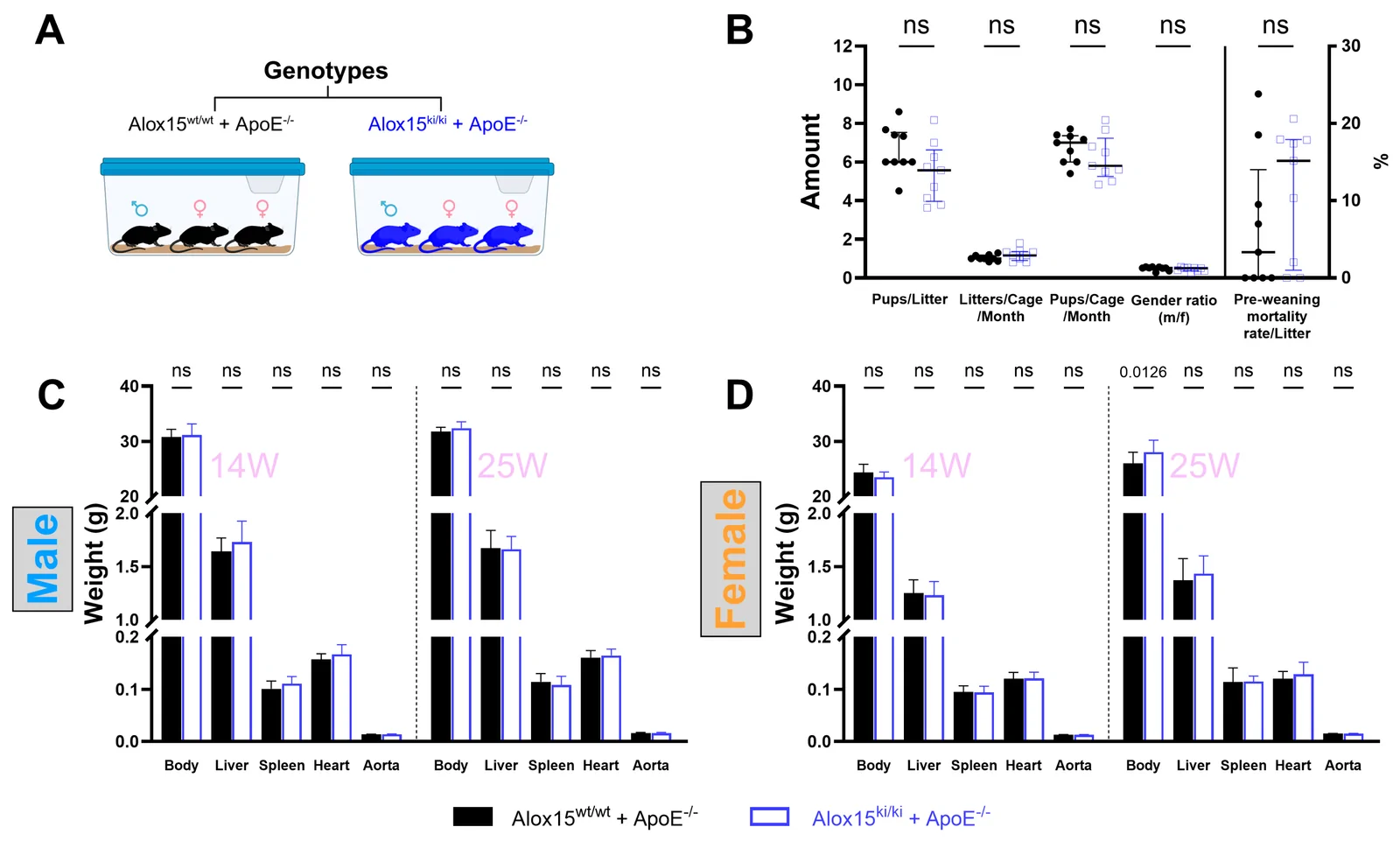
Functional humanization of mouse Alox15 did not induce obvious phenotypic differences. (**A**) Schematic representation of the breeding strategy. Alox15^ki/ki^+ApoE^−/−^ (blue) and Alox15^wt/wt^+ApoE^−/−^ mice (black) were maintained as separate colonies using a one-male-two-female mating system. (**B**) The reproduction behavior was assessed by quantifying five readout parameters: (i) pups per litter, (ii) litters per cage per month, (iii) pups per cage per month, (iv) female-to-male sex ratio, and (v) pre-weaning mortality rate per litter. Data were obtained from nine different Alox15^wt/wt^+ApoE^−/−^ and nine Alox15^ki/ki^+ApoE^−/−^ breeding cages. Median with interquartile range (IQR) is given; Mann–Whitney U test. (**C**) Body and organ weights of 14-week-old and 25-week-old males. (**D**) Body and organ weights of 14-week-old and 25-week-old females; means ± standard deviations are given; n = 15, Student’s *t*-test; ns, not significant.

To rule out the possibility that the in vivo mutagenesis of the mouse Alox15 gene induced major developmental defects, we compared the reproduction behavior (Figure 3A,B) and the body and organ weights (Figure 3C,D) of Alox15ki/ki+ApoE^−/−^ mice with the corresponding parameters of *Alox15^wt/wt^+ApoE^−/−^* control animals.

Here we found major differences neither in the reproduction properties nor in body and organ weights. A modest increase in body weight was observed in 25-week-old female Alox15^ki/ki^+ApoE^−/−^ mice (*p* = 0.0126), but the extent was rather small. We also evaluated the moving behavior, aggressiveness, and fur properties (Figure S5) of the animals. Here again, we did not observe obvious phenotypic differences between the genotypes.

### 2.4. Functional Humanization of Mouse Alox15 Does Not Impact the Degree of Hypercholesterolemia in ApoE-Deficient Mice

**Figure 4:**
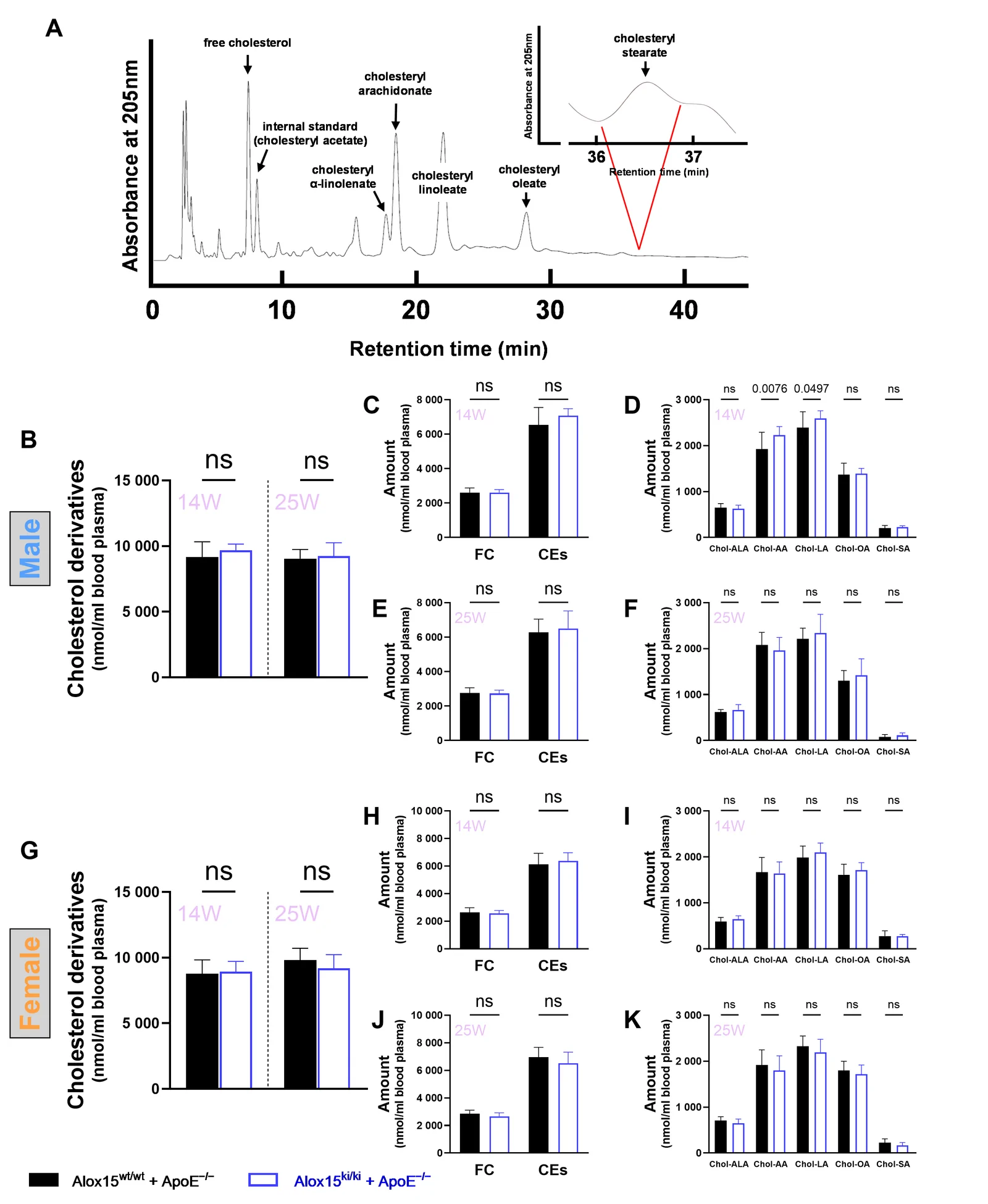
Quantification of plasma cholesterol lipidomes of Alox15^ki/ki^+ApoE^−/−^ mice and Alox15^wt/wt^+ApoE^−/−^ controls. (**A**) Representative RP-HPLC chromatogram of the blood plasma cholesterol lipidomes. (**B**) Total blood plasma cholesterol concentrations (sum of free and esterified cholesterol) of 14- and 25-week-old male Alox15^wt/wt^+ApoE^−/−^ mice (black columns) and Alox15^ki/ki^+ApoE^−/−^ individuals (blue columns). (**C**) Free cholesterol and cholesterol esters concentrations of 14-week-old male Alox15^wt/wt^+ApoE^−/−^ mice (black columns) and Alox15^ki/ki^+ApoE^−/−^ individuals (blue columns). (**D**) Concentrations of the different cholesterol esters of 14-week-old male Alox15^wt/wt^+ApoE^−/−^ mice (black columns) and Alox15^ki/ki^+ApoE^−/−^ individuals (blue columns). (**E**) Free cholesterol and cholesterol esters concentrations of 25-week-old male Alox15^wt/wt^+ApoE^−/−^ mice (black columns) and Alox15^ki/ki^+ApoE^−/−^ individuals (blue columns). (**F**) Concentrations of the different cholesterol esters of 25-week-old male Alox15^wt/wt^+ApoE^−/−^ mice (black columns) and Alox15^ki/ki^+ApoE^−/−^ individuals (blue columns). (**G**) Total cholesterol concentrations (sum of free and esterified cholesterol) in the blood plasma of 14- and 25-week-old female Alox15^wt/wt^+ApoE^−/−^ mice (black columns) and Alox15^ki/ki^+ApoE^−/−^ individuals (blue columns). (**H**) Free cholesterol and cholesterol esters concentrations of 14-week-old female Alox15^wt/wt^+ApoE^−/−^ mice (black columns) and Alox15^ki/ki^+ApoE^−/−^ individuals (blue columns). (**I**) Concentrations of the different cholesterol esters of 14-week-old female Alox15^wt/wt^+ApoE^−/−^ mice (black columns) and Alox15^ki/ki^+ApoE^−/−^ individuals (blue columns). (**J**) Free cholesterol and cholesterol esters concentrations of 25-week-old female Alox15^wt/wt^+ApoE^−/−^ mice (black columns) and Alox15^ki/ki^+ApoE^−/−^ individuals (blue columns). (**K**) Concentrations of the different cholesterol esters of 25-week-old female Alox15^wt/wt^+ApoE^−/−^ mice (black columns) and Alox15^ki/ki^+ApoE^−/−^ individuals (blue columns). Means ± standard deviations are given; n = 14 per genotype for each age and sex group, Student’s *t*-test; ns, not significant.

ApoE-deficient mice develop hypercholesterolemia even when they are kept on standard chow diet [38]. To determine whether functional humanization of Alox15 affects systemic cholesterol metabolism, we quantified the blood plasma cholesterol lipidomes of Alox15^ki/ki^+ApoE^−/−^ mice by RP-HPLC and compared them with those of outbred Alox15^wt/wt^+ApoE^−/−^ controls (Figure 4A).

Because cholesterol derivatives have different molar extinction coefficients at 205 nm, the RP-HPLC system was calibrated before analysis for each cholesterol derivative (Figure S1). In male mice, the total plasma cholesterol concentrations (sum of all quantified cholesterol derivatives in nmol/mL blood plasma) were not significantly different between the two genotypes in either 14-week-old or 25-week-old animals (Figure 4B). Similarly, separate quantification of free cholesterol and the sum of the different cholesterol esters did not reveal significant differences (Figure 4C,E). When we separately quantified the different cholesterol esters, significant differences were observed for cholesteryl arachidonate (*p* = 0.0076) and cholesteryl linoleate (*p* = 0.0497) in 14-week-old animals (Figure 4D). A difference was detected in the 14-week cohort, whereas the corresponding comparisons in the 25-week cohort were not statistically significant (Figure 4F). As in male mice, the total plasma cholesterol concentrations of female individuals were similar in the two genotypes for 14-week-old and 25-week-old individuals (Figure 4G). Similarly, no genotype-specific differences were observed between the two genotypes when the free cholesterol concentrations and the sum of the different cholesterol esters were compared (Figure 4H,J). This was also the case when we compared the cholesterol ester patterns at either time point (Figure 4I,K).

### 2.5. Functional Humanization of Mouse Alox15 Does Not Impact the Lesional Area of the Aorta as Suggested by Morphometric En Face Analysis

**Figure 5:**
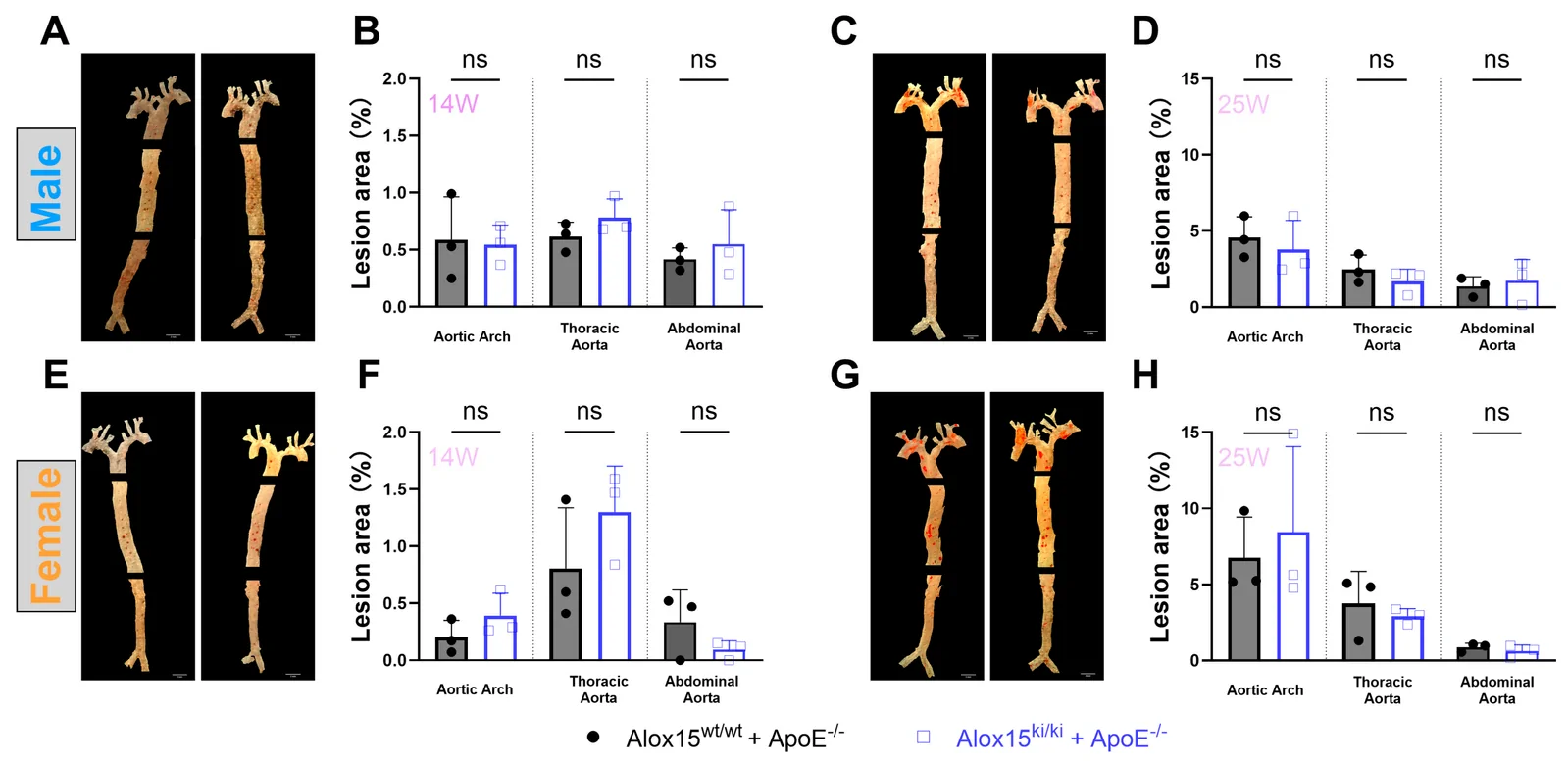
En face Oil Red O staining of the aortic trees prepared from 14-week-old and 25-week-old male and female individuals of Alox15^ki/ki^+ApoE^−/−^ mice and Alox15^wt/wt^+ApoE^−/−^ controls. (**A**) Oil Red O-stained aorta sections, 14-week-old males. (**B**) Morphometric quantification, 14-week-old males. (**C**) Oil Red O-stained aorta sections, 25-week-old males. (**D**) Morphometric quantification, 25-week-old males. (**E**) Oil Red O-stained aorta sections, 14-week-old females. (**F**) Morphometric quantification, 14-week-old females. (**G**) Oil Red O-stained aorta sections, 25-week-old females. (**H**) Morphometric quantification, 25-week-old females. Means ± standard deviations are given; n = 3, Student’s *t*-test; ns, not significant.

To assess arterial lipid deposition, we first quantified the Oil Red O-positive aortic surface area by en face analysis. For this purpose, the aortic sections (aortic arch, thoracic aorta, abdominal aorta) were stained with Oil Red O solution, and the lesional area was quantified morphometrically (Figure 5). The spatial pattern of lesion formation was similar for the two genotypes, but the aortic arch showed the highest degree of plaque formation. The abdominal aorta remained largely unaffected, which is consistent with previous findings [38]. In 14-week-old individuals, the degree of aortic lipid deposition in male (Figure 5A,B) and female (Figure 5E,F) individuals was rather small and varied between 0.1 and 1% of the aortic surface.

In older mice (25-week-old individuals), this readout parameter was higher (Figure 5C,D for males and Figure 5G,H for females), and at this age the lesional areas reached values of up to 5%. These data indicate that the analyzed animals predominantly represented an early stage of vascular lipid deposition. However, functional humanization of Alox15 did not significantly modify overall plaque burden in any anatomical segment of the aorta of either sex.

### 2.6. Functional Humanization of Mouse Alox15 Did Not Impact the Degree of Aortic Lipid Deposition

**Figure 6:**
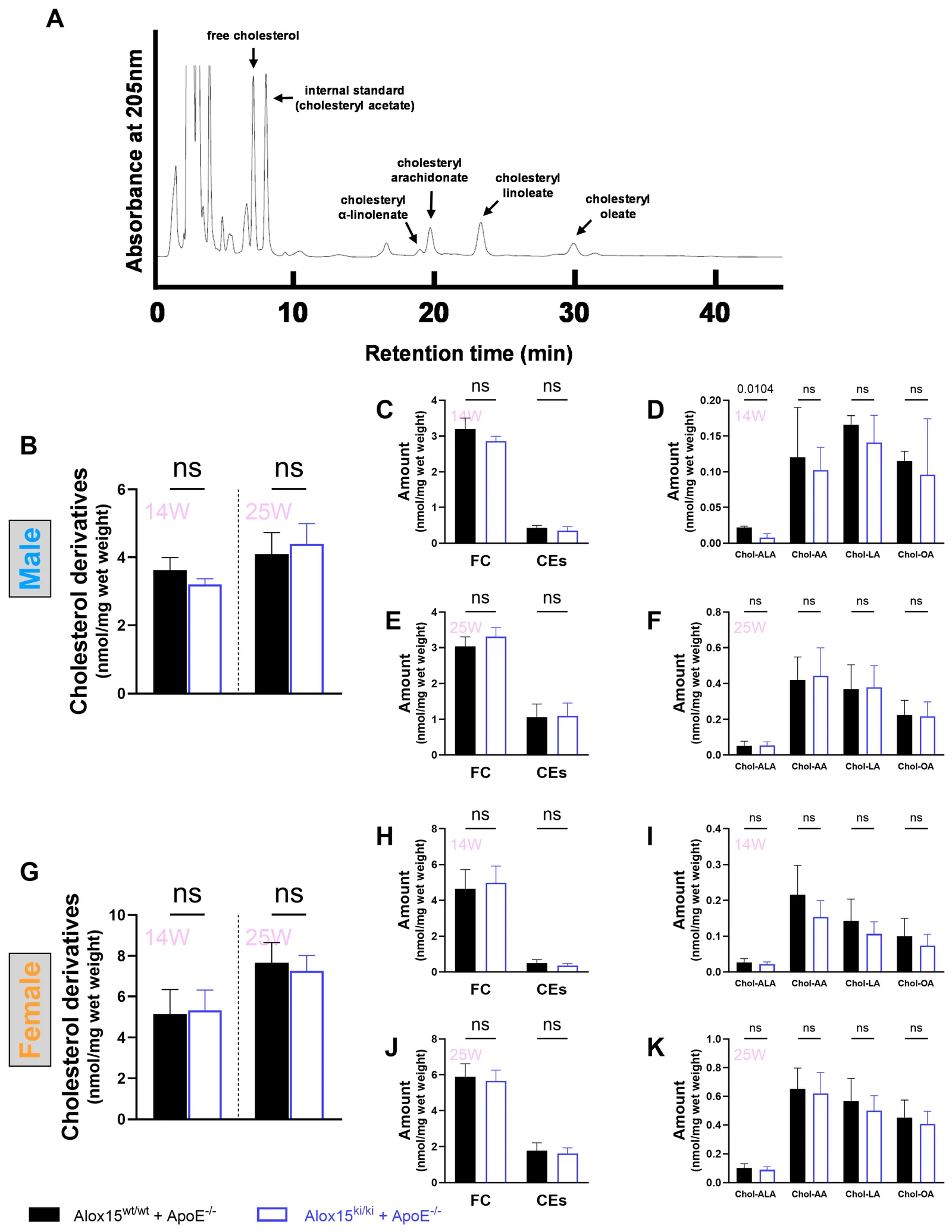
Quantification of the aortic arch cholesterol lipidomes of Alox15^ki/ki^+ApoE^−/−^ mice and Alox15^wt/wt^+ApoE^−/−^ controls. (**A**) Representative RP-HPLC chromatogram of the aortic arch cholesterol lipidomes. (**B**) Total aortic arch cholesterol concentrations (sum of free and esterified cholesterol) of 14- and 25-week-old male Alox15^wt/wt^+ApoE^−/−^ mice (black columns) and Alox15^ki/ki^+ApoE^−/−^ individuals (blue columns). (**C**) Free cholesterol and cholesterol esters concentrations of 14-week-old male Alox15^wt/wt^+ApoE^−/−^ mice (black columns) and Alox15^ki/ki^+ApoE^−/−^ individuals (blue columns). (**D**) Concentrations of the different cholesterol esters of 14-week-old male Alox15^wt/wt^+ApoE^−/−^ mice (black columns) and Alox15^ki/ki^+ApoE^−/−^ individuals (blue columns). (**E**) Free cholesterol and cholesterol esters concentrations of 25-week-old male Alox15^wt/wt^+ApoE^−/−^ mice (black columns) and Alox15^ki/ki^+ApoE^−/−^ individuals (blue columns). (**F**) Concentrations of the different cholesterol esters of 25-week-old male Alox15^wt/wt^+ApoE^−/−^ mice (black columns) and Alox15^ki/ki^+ApoE^−/−^ individuals (blue columns). (**G**) Total aortic arch cholesterol concentrations (sum of free and esterified cholesterol) of 14- and 25-week-old female Alox15^wt/wt^+ApoE^−/−^ mice (black columns) and Alox15^ki/ki^+ApoE^−/−^ individuals (blue columns). (**H**) Free cholesterol and cholesterol esters concentrations of 14-week-old female Alox15^wt/wt^+ApoE^−/−^ mice (black columns) and Alox15^ki/ki^+ApoE^−/−^ individuals (blue columns). (**I**) Concentrations of the different cholesterol esters of 14-week-old female Alox15^wt/wt^+ApoE^−/−^ mice (black columns) and Alox15^ki/ki^+ApoE^−/−^ individuals (blue columns). (**J**) Free cholesterol and cholesterol esters concentrations of 25-week-old female Alox15^wt/wt^+ApoE^−/−^ mice (black columns) and Alox15^ki/ki^+ApoE^−/−^ individuals (blue columns). (**K**) Concentrations of the different cholesterol esters of 25-week-old female Alox15^wt/wt^+ApoE^−/−^ mice (black columns) and Alox15^ki/ki^+ApoE^−/−^ individuals (blue columns). Means ± standard deviations are given; n = 3 per genotype for 14-week-old males, n = 6 per genotype for 14-week-old females, n = 7 per genotype for 25-week-old males and females, Student’s *t*-test.

En face analysis (Figure 5) quantified the area of lipid deposits in relation to the unaffected arterial surface. This value is a robust measure but may not be very sensitive. To determine a second readout parameter for the degree of aortic lipid deposition, we extracted the total lipids from the different aortic segments and quantified the tissue cholesterol lipidomes (Figure 6). In Figure 6A, a representative RP-HPLC chromatogram of the total lipid extracts of the aortic arch tissue is shown. As for the extracts of the blood plasma lipids (Figure 4A), the different cholesterol derivatives were well resolved and could be quantified separately. The tissue concentrations of cholesteryl stearate were below the detection limits of our RP-HPLC system, and thus we refrained from quantifying this particular readout parameter.

In male mice, the aortic arch tissue levels of the total cholesterol derivatives (sum of free and esterified cholesterol) did not differ significantly between the two genotypes in either 14-week-old or 25-week-old mice (Figure 6B). Similarly, separate quantification of the tissue concentrations of free cholesterol and the total cholesterol esters did not reveal genotype-specific differences (Figure 6C,E). Separate quantification of the aortic arch tissue concentrations of the different cholesterol esters revealed elevated tissue levels of cholesteryl-alpha-linolenate (chol-ALA) in 14-week-old Alox15^wt/wt^+ApoE^−/−^ mice (control animals) when compared with Alox15^ki/ki^+ApoE^−/−^ animals (experimental group, *p* = 0.0104; Figure 6D). For all other cholesterol esters, no significant genotype-specific differences were observed. For 25-week-old individuals (Figure 6E,F), no significant genotype-specific differences were detected for any cholesterol derivative. A difference in cholesteryl-alpha-linolenate (chol-ALA) tissue concentrations was detected between the two genotypes in 14-week-old males, whereas the corresponding comparison in 25-week-old males was not statistically significant.

In female mice, aortic arch lipid analyses revealed no statistically significant genotype-specific differences in 14-week-old and in 25-week-old animals (Figure 6G). Free cholesterol and cholesterol ester fractions were comparable between Alox15^ki/ki^+ApoE^−/−^ and Alox15^wt/wt^+ApoE^−/−^ mice (Figure 6H,J), and no genotype-specific differences were observed in the patterns of aortic cholesterol ester sub-species (Figure 6I,K).

In summary, it was concluded that functional humanization of mouse Alox15 does not induce significant differences in aortic lipid deposition. Subtle differences in the vascular patterns of individual cholesterol esters were small and transient and thus may not be of biological relevance.

### 2.7. Functional Humanization of Mouse Alox15 Did Not Consistently Impact the Expression of Inflammation-Related Gene Products

Since atherosclerosis is an inflammatory disease [21,39], we next quantified the expression levels of classical pro- and anti-inflammatory gene products in the wall of the aortic arch of 14- and 25-week-old mice of either sex. For this purpose, we extracted total RNA from the aortic tissue and quantified the steady-state concentrations of the mRNAs encoding the following pro-inflammatory proteins: (i) inducible nitric oxide synthase (iNOS) [40], (ii) tumor necrosis factor alpha (TNF_alpha_) [41], and (iii) prostaglandin synthase 2 (PTGS2), which is commonly known as cyclooxygenase 2 (COX2) [42]. In parallel, we also quantified the expression of anti-inflammatory/pro-resolving gene products: (i) transforming growth factor beta (TGF_beta_) [43], (ii) interleukin 10 (IL10) [44], and (iii) arginase-1 (Arg1) [45], which plays a major role in alternative polarization of pro-resolving M2 macrophages [46].

**Figure 7:**
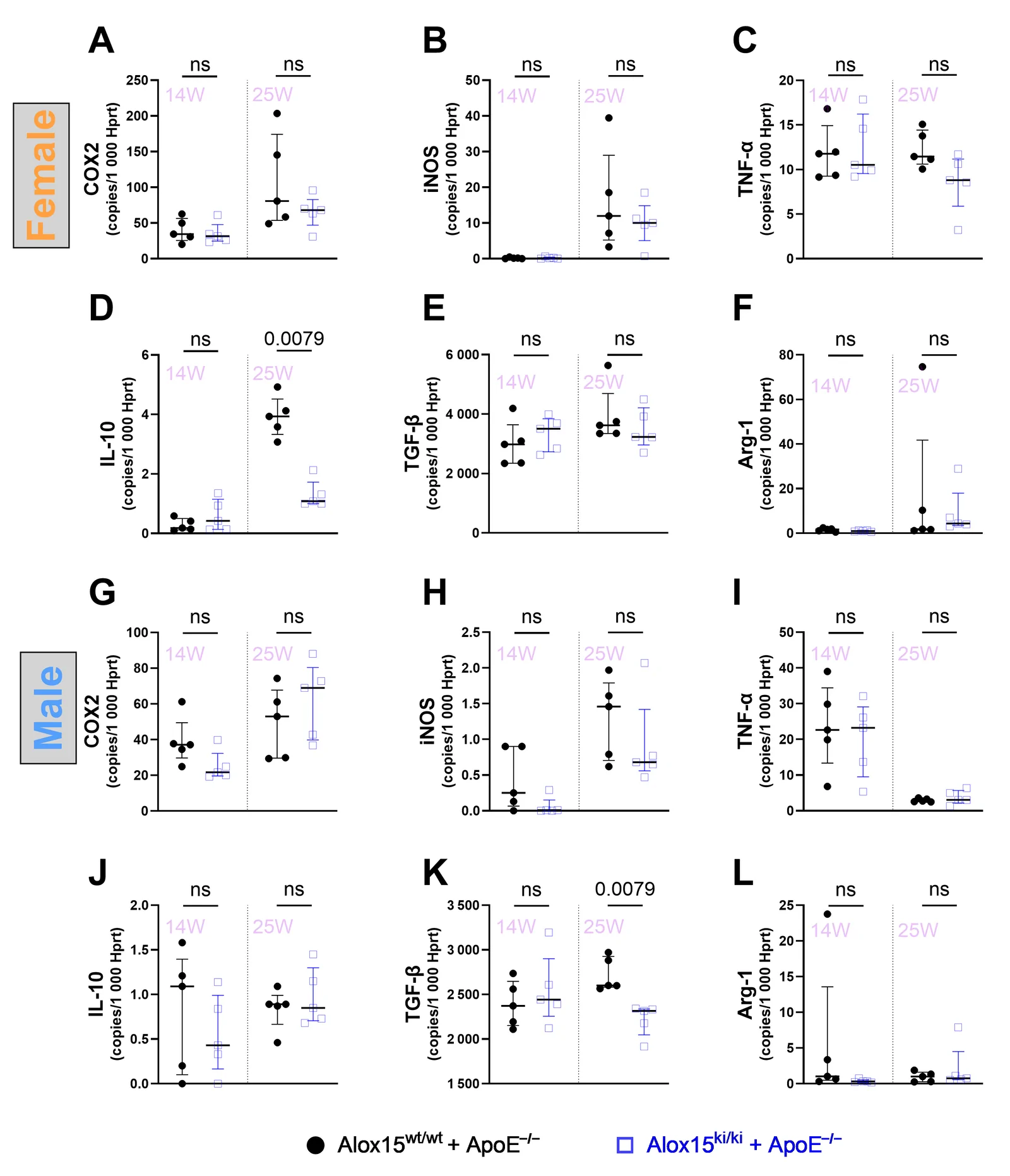
Expression levels of classical pro- and anti-inflammatory gene products in the wall of the aortic arch of 14- and 25-week-old Alox15^ki/ki^+ApoE^−/−^ mice and Alox15^wt/wt^+ApoE^−/−^ controls of either sex. Total RNA was extracted from the aortic arch and was reverse transcribed. The target cDNA species were amplified using gene-specific primers (Table S2). cDNA preparations of five independent individuals were amplified, and each sample was run in triplicate. (**A**–**C**) Pro-inflammatory gene products, females. (**D**–**F**) Anti-inflammatory gene products, females. (**G**–**I**) Pro-inflammatory gene products, males. (**J**–**L**) Anti-inflammatory gene products, males. Median with interquartile range (IQR) is given; n = 5, Mann–Whitney U test.

In female individuals, we did not find significant differences in the expression levels of pro-inflammatory gene products (Figure 7A–C) between Alox15^ki/ki^+ApoE^−/−^ mice (experimental group) and outbred wild-type control animals (Alox15wt/wt+ApoE-/-), and similar results were obtained when the expression levels of anti-inflammatory/pro-resolving gene products were compared (Figure 7D,F). Although we observed a genotype-specific difference in lesional IL10 expression for 25-week-old females (Figure 7D), the extent of this difference was not dramatic and thus may not be of biological relevance. For male individuals (Figure 7G,L), the expression levels of most analyzed genes were comparable for the two genotypes. However, TGFbeta transcript abundance was significantly different in 25-week-old males (*p* = 0.0079; Figure 7K). Taken together, this data indicates that the expression profile of the selected inflammation-related genes was largely similar in the two genotypes, although isolated genotype-specific differences were observed.

## 3. Discussion

In different mammals (humans, mice, rabbits), ALOX15 orthologs have been implicated in atherogenesis, but the precise biological role of the enzyme is still a matter of discussion [23,47]. In humans, which express an AA 15-lipoxygenating ALOX15 ortholog [18], epidemiological studies have previously related different polymorphisms in the ALOX15 gene to human atherosclerosis [24,25,26,27], but controversial results have been reported. Specific ALOX15 products (13S-HODE) have been detected in young and advanced human atherosclerotic lesions [48,49], and these data suggested the in vivo activity of the enzyme in the early stages of atherogenesis. At later stages, non-enzymatic lipid peroxidation products apparently become more relevant [48,49]. In rabbits, which also express an AA 15-lipoxygenating ALOX15 ortholog [50], a similar pattern of specific ALOX15 products has been detected in atherosclerotic lesions [51], and in this model overexpression of ALOX15 reduced the atherosclerotic burden. Overexpression of human ALOX15 under the control of a macrophage-specific promoter protected transgenic rabbits from arterial lipid deposition, and thus the enzyme appeared to exhibit anti-atherogenic activities [33]. This gain-of-function strategy (overexpression of human ALOX15 in rabbits) was plausible, since human and rabbit ALOX15 orthologs have similar functional properties. In mice, which express an AA 12-lipoxygenating Alox15 ortholog, the enzyme has also been implicated in atherogenesis, but in this species controversial results have been reported in a large number of loss-of-function and gain-of-function studies [28,30,31,34,52]. The possible reasons for the controversial outcomes of the different studies have been discussed, but one aspect has frequently been ignored. Human and rabbit ALOX15 orthologs are AA 15-lipoxygenating enzymes [18,50]. In contrast, mouse Alox15 constitutes an AA 12-lipoxygenating enzyme [17,53]. This functional difference not only affects the patterns of single oxygenation products but also the more complex mixtures of secondary fatty acid oxygenation products such as pro-inflammatory hepoxilins [5] and various specialized pro-resolving mediators (SPMs) [54]. Although the biological relevance of SPMs has recently been challenged [55], their biosynthesis strongly depends on the reaction specificity of the enzymes. In fact, AA 12-lipoxygenating ALOX15 orthologs form different patterns of SPMs than AA 15-lipoxygenating orthologs.

Because mouse and human ALOX15 orthologs have distinct catalytic properties, translation of murine findings to humans is difficult. Humanized Alox15^ki/ki^ mice expressing the AA 15-lipoxygenating Leu353Phe mutant were therefore generated [19] and shown to be protected from intestinal inflammation [20]. Here, we crossed these mice into an ApoE^−/−^ background and characterized the degree of aortic lipid deposition and the expression patterns of selected inflammation-related gene products as the major readout parameters. Unfortunately, we found consistent genotype-specific differences neither in the extent of aortic lipid deposition nor in the vascular expression patterns of the selected gene products. Despite this “negative outcome”, the data are of biological relevance since our results suggest that early vascular lipid deposition under these experimental conditions does not depend on the positional specificity of Alox15. In other words, the reaction specificity of Alox15 may not be of major patho-physiological relevance.

A critical point of our studies is the limited n-numbers. The impact of functional humanization of Alox15 reaction specificity on atherogenesis has not been investigated before, and thus reliable prospective sample-size calculations could not be carried out. Altogether, we included 120 mice in the study and distributed them across different experimental groups defined by age, sex, and genotype. Since multiple readout parameters (lipid lesional area, aortic cholesterol lipidomes, vascular gene expression patterns) were analyzed and since simultaneous quantification of these parameters from a single aortic segment was not possible, the number of biological replicates available for individual quantification was smaller than expected. In fact, the n-numbers in different experimental groups varied because of preparation artifacts and/or technical failures.

In order to quantify the degree of vascular inflammation, we quantified the vascular expression of selected pro- and anti-inflammatory gene products by qRT-PCR, but this data does not allow reliable quantification of the extent of vascular inflammation. Other readout parameters, such as the presence of the corresponding proteins, formation of inflammatory edema, or invasion of inflammatory cells, would have quantified the degree of vascular inflammation more comprehensively. However, since we did not see major genotype-specific differences in our qRT-PCR studies, we refrained from performing corresponding analyses.

Another limitation of our study is that the impact of humanized Alox15 reaction specificity on atherosclerosis was investigated in one particular mouse model (ApoE^−/−^ mice). Other mouse atherosclerosis models, such as LDL-receptor-deficient mice [56] or disturbed-flow-induced atherosclerosis models [28,32], have not been tested. Moreover, since we were most interested in the early stages of lesion development, we kept the animals on standard chow diet for a short time period (up to 25 weeks). Thus, our data do not address mechanistic aspects of later stages of plaque development. Therefore, our results do not exclude the possibility that functional humanization of mouse Alox15 might induce different vascular effects in other mouse atherosclerosis models or at more advanced stages of lesion development. However, since Alox15^ki/ki^ mice are freely available for collaborative research, corresponding experiments can easily be carried out in the future.

## 4. Materials and Methods

### 4.1. Chemicals

The major chemicals used in this study were from the following sources: Arachidonic acid (AA), 15R/S-HETE, 12R/S-HETE, and 8R/S-HETE were purchased from Biomol GmbH (Hamburg, Germany). Free cholesterol was obtained from Serva Electrophoresis GmbH (Heidelberg, Germany), cholesteryl acetate from Santa Cruz Biotechnology (Dallas, TX, USA), cholesteryl stearate, cholesteryl oleate, cholesteryl linoleate, and cholesteryl arachidonate from Merck (Taufkirchen, Germany), and cholesteryl linolenate from MedChemExpress (Monmouth Junction, NJ, USA). Oil Red O was purchased from Merck KGaA (Taufkirchen, Germany), sodium borohydride from Life Technologies, Inc. (Eggenstein, Germany), and isopropyl β-D-1-thiogalactopyranoside (IPTG) from Carl Roth GmbH (Karlsruhe, Germany). Restriction endonucleases were obtained from Thermo Fisher Scientific, and *Escherichia coli* Rosetta 2 (DE3) pLysS cells from Novagen (Darmstadt, Germany). HPLC-grade acetonitrile, 2-propanol, water, n-hexane, acetic acid, methanol, ethanol, and chloroform were purchased from Fisher Scientific GmbH (Schwerte, Germany). DPBS was obtained from PAN-Biotech (Aidenbach, Germany). For immunoblotting of the recombinant ALOX isoforms, an anti-hexa-his-tag antibody (Miltenyi Biotech & Co. KG, Bergisch-Gladbach, Germany) was used at a 1:5000 dilution. All other chemicals and their commercial sources are summarized in Table S1 in the Supplementary Material.

### 4.2. Bacterial Expression of Recombinant Alox15 Variants

Mouse Alox15 variants were expressed as N-terminal hexa-histidine-tagged fusion proteins in *Escherichia coli* Rosetta 2 (DE3) pLysS cells as described in [37] with minor modifications. A more detailed description of the expression procedure is given in the Methodological Supplement (Chapter S2.1). Bacterial cultures were grown in the presence of kanamycin (50 µg/mL) and chloramphenicol (35 µg/mL). Recombinant protein expression was induced with 1 mM isopropyl β-D-1-thiogalactopyranoside (IPTG), and cultures were subsequently incubated at 22 °C for 18 h. Bacteria were harvested by centrifugation, resuspended in DPBS, and disrupted by sonication. Cell debris was removed by centrifugation (15,000× *g*, 15 min, 4 °C) and the cell-free bacterial lysate supernatants were used as enzyme source for functional characterization.

### 4.3. In Vitro Arachidonic Acid Oxygenase Activity Assays

Catalytic activity of the recombinant ALOX15 preparations was assessed using arachidonic acid (AA) as substrate as described in [37] with minor modifications. A detailed description of the activity assays is given in the Methodological Supplement (Chapter S2.2). Aliquots of the enzyme preparations were incubated for 3 min at room temperature in 0.5 mL DPBS containing 100 µM AA. The reaction was terminated by addition of 1 mg sodium borohydride (NaBH_4_), which simultaneously reduced the hydroperoxy fatty acid products to the corresponding hydroxy derivatives. After 5 min on ice, 75 µL glacial acetic acid and 0.5 mL acetonitrile were added, and precipitated proteins were removed by centrifugation. The protein-free supernatants were analyzed by RP-HPLC using a Nucleodur C18 column with acetonitrile/water/acetic acid (70:30:0.1, *v*/*v*/*v*) as the mobile phase at a flow rate of 1 mL/min. Conjugated dienes were monitored at 235 nm. Product formation was quantified using a calibration curve generated with known amounts of 15-HETE.

### 4.4. In Vitro Lipoprotein Oxygenase Activity Assay

Human lipoproteins (LDL, HDL) were prepared by floating ultracentrifugation [57]. Purified lipoproteins were diluted in DPBS to a final protein concentration of 1 mg/mL [37] and incubated with recombinant ALOX15 preparations for 15 min at room temperature as described in [37] with minor modifications, and a detailed description of LDL/HDL preparation as well as of the assay system is given in the Methodological Supplement (Chapter S2.3). The reactions were terminated by addition of 1 mg sodium borohydride, followed by acidification with glacial acetic acid. Total lipids were extracted with methanol and chloroform, and the organic phase was recovered after phase separation. After evaporation of the solvent, the remaining lipids were reconstituted in 350 µL methanol, hydrolyzed under alkaline conditions, and the resulting free fatty acid derivatives were analyzed by RP-HPLC on a Nucleodur C18 column. A mixture of acetonitrile/water/acetic acid (70:30:0.1, *v*/*v*/*v*) was used as the mobile phase at a flow rate of 1 mL/min. Conjugated dienes were monitored at 235 nm and non-oxidized PUFAs were recorded at 210 nm. To quantify the degree of oxygenation of the lipoprotein lipids, we calculated the OH-PUFA/PUFA ratio [37].

### 4.5. Ex Vivo Alox15 Activity Assays

Peritoneal lavage cells were prepared from sacrificed Alox15^ki/ki^+ApoE^−/−^ mice and from Alox15^wt/wt^+ApoE^−/−^ control animals by injecting 10 mL DPBS into the peritoneal cavity as described in [19] with minor modifications. The cells were washed three times in DPBS, and aliquots of the cell suspension containing 2–5 × 10^6^ cells/mL were incubated for 15 min at room temperature in 0.5 mL DPBS containing 100 µM arachidonic acid. A detailed description of the ex vivo activity assay including RP-HPLC-based quantification of the Alox15 products is given in the Methodological Supplement (Chapter S2.4). This description also involves analysis of the reaction products by combined normal phase/chiral phase HPLC (NP/CP-HPLC).

### 4.6. Animals and Breeding Procedure

For the in vivo experiments, the following genetically modified mouse models were employed: (i) ApoE^−/−^ mice, (ii) Alox15^−/−^ mice, and (iii) Alox15^ki/ki^+ApoE^−/−^ mice. More detailed information on these genetically modified mouse strains, including their genetic background, is given in Table S2 in the Supplementary Material.

Homozygous Alox15 knock-in mice (Alox15^ki/ki^, C57BL/6J background) [53] were first crossed with apolipoprotein E-deficient mice (ApoE^−/−^, B6.129P2-Apoetm1Unc/J, Institute of Experimental Medicine, Charite - Universitätsmedizin Berlin, Germany). Double heterozygous offspring were intercrossed, and homozygous Alox15^ki/ki^+ApoE^−/−^ mice as well as ApoE-deficient control animals (Alox15^wt/wt^+ApoE^−/−^) were selected. Separate colonies of mice of both genotypes were bred from these founder animals. Animals from the two genotype-specific colonies were randomly assigned to the different experimental groups according to their genotype, sex, and age. For each genotype, 15 males and 15 females were included at 14 weeks and another 15 males and 15 females at 25 weeks, resulting in a total of 120 mice. Littermate controls were not used; instead, age- and sex-matched Alox15^wt/wt^+ApoE^−/−^ mice from the corresponding control colony served as controls. Within each genotype and sex, animals were randomly assigned to the 14-week or 25-week sampling cohort. Mice were housed under a 12-h light/dark cycle with ad libitum access to standard chow diet and water. All animal experiments were approved by the institutional animal care committee (T-CH0003/22). More detailed information on the in vivo studies (n-numbers, gender, and age distribution) is given in Table S3.

### 4.7. DNA Extraction and Genotyping

Genomic DNA was extracted from ear biopsies and used as a template for PCR amplification of the Alox15 and ApoE gene loci, and a detailed description of the PCR including the sequences of the amplification primers and the Alox15 sequencing primer is given in the Supplementary Material (Table S4). Genomic PCR was initiated by a DNA denaturation step at 95 °C for 15 min, followed by 29 PCR cycles each consisting of a 20 s denaturation step (95 °C), a 30 s annealing step (65 °C), and a 60 s synthesis step (72 °C). After the 29 amplification cycles, the mixture was kept at 72 °C for 10 min and then cooled down to 10 °C. The amplification products were separated on 2% agarose gels. For genotyping of the Alox15 locus, the PCR products were purified using a PCR Clean-up Kit (Macherey-Nagel, Düren, Germany) and subsequently sequenced to distinguish the wild-type and knock-in alleles. For genotyping of the ApoE locus, allele-specific primer combinations were used to discriminate between the wild-type and knockout alleles.

### 4.8. Sample Collection for the In Vivo Experiments and Sample Storage

Mice were anesthetized with isoflurane until loss of the pedal withdrawal reflex and euthanized by cervical dislocation. Blood was collected by cardiac puncture and centrifuged (3000× *g*, 15 min, 4 °C) to recover plasma. The vasculature was then perfused with 10 mL DPBS via the left ventricle. The aorta was prepared, and the vessel was divided into: (i) aortic arch, (ii) thoracic aorta, and (iii) abdominal aorta. The tissue specimens used for lipid extraction were shock-frozen (liquid nitrogen) and stored at −80 °C. For en face Oil Red O staining, aortic segments were fixed in 4% paraformaldehyde. Tissue specimens employed for mRNA extraction were incubated overnight in RNAlater (Sigma-Aldrich, Merck KG, Taufkirchen, Germany) at room temperature, frozen in liquid nitrogen and then stored at −80 °C. Animals from the two different mouse colonies (Alox15^wt/wt^+ApoE^−/−^ vs. Alox15^ki/ki^+ApoE^−/−^) were randomly allocated to the age and sex groups. However, the actual n-numbers in each experimental group were not identical since some tissue samples were lost because of technical problems in sample preparation and/or in sample workup. The exact n-numbers for the different experimental groups are reported in the corresponding figure legends.

### 4.9. Total Lipid Extraction from Blood Plasma and from Aortic Tissue

Total lipids were extracted from blood plasma and aortic arch tissue using a methanol/chloroform extraction procedure based on the method of Bligh and Dyer [58] with minor methodological modifications. A detailed description of the extraction procedure is given in the Methodological Supplement (Chapter S2.5). For plasma samples, 100 µL plasma was diluted with 0.5 mL DPBS and supplemented with 100 µL of an ethanolic cholesteryl acetate solution (0.4 mg/mL), which served as an external extraction standard. Methanol (1.5 mL) and chloroform (0.75 mL) were added, and the samples were vortexed thoroughly. After incubation on ice, an additional 0.75 mL chloroform and 0.75 mL DPBS were added to induce phase separation. Following centrifugation at 5000× *g* for 5 min, the lower organic phase involving the total tissue lipids was recovered. The solvents were evaporated, and the remaining lipids were reconstituted in 100 µL 2-propanol followed by the addition of 300 µL acetonitrile.

For extraction of aortic arch lipids, tissue specimens were homogenized in 1.5 mL methanol after addition of the cholesteryl acetate extraction standard. The homogenates were mixed with 0.6 mL DPBS and 0.75 mL chloroform and vortexed thoroughly. After 10 min incubation on ice, 0.75 mL chloroform and 0.75 mL DPBS were added, and phase separation was achieved by centrifugation at 5000× *g* for 5 min. The lower organic phase was recovered, evaporated to dryness, and the remaining lipids were reconstituted in 100 µL 2-propanol and 300 µL acetonitrile. Lipid extracts were stored at −20 °C under an argon atmosphere until RP-HPLC analysis.

### 4.10. RP-HPLC-Based Quantification of the Cholesterol Lipidomes

For quantification of the cholesterol lipidomes, aliquots of the total lipid extracts were analyzed by RP-HPLC. Cholesterol derivatives were separated on a Nucleodur C18 ec column (250 × 4 mm, 5 µm; Macherey-Nagel GmbH & Co. KG, Düren, Germany) using an isocratic mobile phase consisting of 65% acetonitrile and 35% 2-propanol (0.1% acetic acid) at a flow rate of 1 mL/min and a column temperature of 45 °C. Free cholesterol and individual cholesterol esters were identified by comparison of their retention times with those of authentic standards. Quantification was performed using compound-specific calibration curves (Figure S1). Cholesteryl acetate was used as an external extraction standard to account for sample recovery. Cholesterol concentrations were expressed as nmol/mL plasma or nmol/mg aortic tissue wet weight. The calibration parameters are summarized in Table S6.

### 4.11. En Face Analysis of Aortic Lipid Deposition

Specimens of the aortic trees were fixed in 4% PFA at 4 °C. The aorta was placed in a 1.5 mL Eppendorf tube and washed three times with 1 mL of 78% methanol (MeOH). Then, 1 mL of freshly prepared Oil Red O solution (0.2% ORO in a 5:2 mixture of methanol and 1 M NaOH) was added, and the samples were incubated at room temperature for 60 min. The stained samples were transferred to clean Eppendorf tubes and were washed twice with 1 mL of 78% MeOH. Following the washes, the aorta samples were placed on a dark dissecting plate, the remaining connective tissue was removed, and the percentage of ORO-positive surface area was quantified.

### 4.12. Extraction of Total RNA from Aortic Tissue and qRT-PCR

To quantify the vascular steady-state transcript concentrations of inflammation-related gene products, we quantified the number of mRNA copies of three classical pro-inflammatory gene products [inducible nitric oxide synthase (iNOS), prostaglandin synthase 2 (Ptgs2, COX2), tumor necrosis factor (Tnf_alpha_)] and three anti-inflammatory/pro-resolving genes [transforming growth factor-beta (Tgf_beta_), arginase-1 (Arg1), interleukin-10 (Il10)] in the aortic arch of 14- and 25-week-old mice of either genotype and sex using a strictly quantitative qRT-PCR approach. This method involved the cloning of external amplification standards for each target gene (iNos, Ptgs2, Tnfalpha, Tgf_beta_, Arg1, IL10) and for the reference gene (Hprt). Target mRNA copy numbers were normalized to 1000 copies of the reference mRNA.

Aortic arch tissue was preserved in RNAlater immediately after removal from the sacrificed animals. After thawing, the tissue was minced and homogenized in 700 µL TRI Reagent using a FastPrep-24 homogenizer. Chloroform (140 µL) was added, and after phase separation by centrifugation at 12,000× *g* for 15 min at 4 °C, the aqueous phase was recovered and mixed with an equal volume of 70% ethanol. Total RNA was subsequently purified using the RNeasy Mini Kit (Qiagen GmbH, Hilden, Germany) according to the manufacturer’s instructions and eluted in 30 µL RNase-free water. RNA concentration and purity were assessed spectrophotometrically by quantifying the A260/A280 ratio. For reverse transcription, RNA samples were adjusted to 40 ng/µL, and 12.5 µL of the RNA preparation was combined with 7.5 µL reverse transcription mixture. Reverse transcription was performed for 60 min at 45 °C, followed by enzyme inactivation for 5 min at 85 °C. The resulting cDNA was stored at −20 °C until analysis.

qRT-PCR was performed using 1 µL cDNA, 5 µL 2× SensiFAST SYBR No-ROX Mix, and 4 µL water. After an initial denaturation step at 95 °C for 10 min, 40 amplification cycles were run, each consisting of 15 s at 95 °C, 30 s at 65 °C, and 20 s at 72 °C. For precise quantification of the number of mRNA copies in the aortic tissue, external amplification standards were prepared for each target gene and the reference gene (*Hprt*). For this purpose, we amplified a representative intron-spanning piece of the corresponding cDNA and cloned it into a high-copy-number cloning plasmid. The recombinant plasmids were then prepared, linearized and used as external amplification standards. Plasmid concentrations were determined by UV spectroscopy and converted to copy numbers per µL based on their molecular weight. Serial dilutions of the amplification standard stock solutions were carried out, and calibration curves for each target gene and for the internal reference gene (*Hprt*) were established. No-template controls were included in every qPCR run. Melting curves were recorded online for each sample to ensure homogeneity of the amplification products. Since vascular Gapdh expression was regulated, we selected the hypoxanthine guanine phosphoribosyltransferase (Hprt) as the reference gene [59]. The nucleotide sequences of the gene-specific amplification primers are given in the Supplementary Material (Table S5).

### 4.13. Experimental Repetitions and Statistical Evaluation of the Raw Data

Experimental repetitions (n-numbers) are specified in the figure and table legends. Statistical analyses were performed using GraphPad Prism version 10.1.2 (Build 324). Normally distributed data were analyzed using Student’s *t*-test or Welch’s *t*-test when variances were unequal; non-normally distributed data were analyzed using the Mann–Whitney U test. *p* values < 0.05 were considered statistically significant.

## 5. Conclusions

Humanization of the reaction specificity of mouse Alox15 induced consistent genotype-specific differences neither in early aortic lipid deposition nor in the vascular expression patterns of selected inflammation-related gene products. Thus, the reaction specificity of Alox15 may not be of major importance for early atherogenesis in ApoE-deficient mice.

## Data Availability

The original experimental raw data can be obtained upon request from H.K. and J.Y.

## Supplementary Materials

The following supporting information can be downloaded at: https://www.mdpi.com/article/10.3390/ijms27219480/s1.

## Abbreviations

The following abbreviations were used in this manuscript. ALOX, arachidonic acid lipoxygenase; PUFA, polyenoic fatty acid; LA, linoleic acid; AA, arachidonic acid; DHA, docosahexaenoic acid; EPA, eicosapentaenoic acid; GLA, gamma-linolenic acid; ALA, alpha-linolenic acid; 15-H(p)ETE, 15-hydro(pero)xy-5Z,8Z,11Z,13E-eicosatetraenoic acid; 12-H(p)ETE, 12-hydro(pero)xy-5Z,8Z,10E,14Z-eicosatetraenoic acid; 13-H(p)ODE, 13-hydro(pero)xy-9Z,11E-octadecadienoic acid; MeOH, methanol; ApoE, Apolipoprotein E; DPBS, Dulbecco’s phosphate-buffered saline; Arg1, arginase-1; OH-PUFA/PUFA ratio, hydroxy polyunsaturated/polyunsaturated fatty acid ratio; HPLC, high-performance liquid chromatography; RT-PCR, reverse transcriptase polymerase chain reaction; Tgf_β_, transforming growth factor beta; iNos, inducible nitric oxide synthase; Tnf_α_, tumor necrosis factor alpha; Hprt, hypoxanthine guanine phosphoribosyltransferase; LDL, low-density lipoprotein; HDL, high-density lipoprotein.
