# Supplementary material for: Functional Humanization of Mouse Alox15 Does Not Alter Early Vascular Lipid Deposition in ApoE-Deficient Mice
Source: Int J Mol Sci. 2026 Oct 23;27(21):9480. doi: 10.3390/ijms27219480 (PMC13762508; doi:10.3390/ijms27219480)
Supplement: Supplementary file 1 [file ijms-27-09480-s001.zip › ijms-4611081-supplementary.pdf]

Supplementary material to the paper

Humanization of the reaction specificity of mouse Alox15 does not alter early vascular lipid deposition in ApoE-deficient mice.

Jiaxing Yang[1], Astrid Borchert[1], Dilyana Branimirova Mangarova[2], Zixiao Zhao[3], Bowen Wang[4] and Hartmut Kühn[1]*

[1]Charité - Universitätsmedizin Berlin, Corporate member of Freie Universität Berlin and Humboldt Universität zu Berlin, Department of Biochemistry, Charitéplatz 1, D-10117 Berlin, Germany.

[2]Charité - Universitätsmedizin Berlin, Corporate member of Freie Universität Berlin and Humboldt Universität zu Berlin, Department of Radiology, Charitéplatz 1, D-10117 Berlin, Germany

[3]Charité – Universitätsmedizin Berlin, Corporate member of Freie Universität Berlin and Humboldt Universität zu Berlin, Clinical Research Center for Hair and Skin Science, Department of Dermatology and Allergy, Charitéplatz 1, 10117 Berlin, Germany.

[4]The First Affiliated Hospital of Harbin Medical University, Urology Surgery Department, 37 Youzheng Street, Nangang District, 150001 Harbin, China.

Short title: ALOX15 humanization and atherogenesis

Funding sources: Financial support for this research was declined by Deutsche Forschungsgemeinschaft (Ku 961/17-1) but funded by the China Scholarship Council (grant No. 202208080179).

*Address correspondence to: Hartmut Kuhn (hartmut.kuehn@charite.de)

1. Resource tables

1.1. Chemicals and their commercial sources (**Table S1**)

Description	Source / Repository
Acetonitrile (HPLC grade)	Fisher Scientific GmbH, Schwerte, Germany
Isopropanol (2-propanol, HPLC grade)	Fisher Scientific GmbH, Schwerte, Germany
Water (HPLC grade)	Fisher Scientific GmbH, Schwerte, Germany
n-Hexane (HPLC grade)	Fisher Scientific GmbH, Schwerte, Germany
Acetic acid (HPLC grade)	Fisher Scientific GmbH, Schwerte, Germany
Methanol (HPLC grade)	Fisher Scientific GmbH, Schwerte, Germany
Ethanol (HPLC grade)	Fisher Scientific GmbH, Schwerte, Germany
Chloroform (HPLC grade)	Fisher Scientific GmbH, Schwerte, Germany
Arachidonic acid	Biomol GmbH, Hamburg, Germany
15R/S-HETE	Biomol GmbH, Hamburg, Germany
12R/S-HETE	Biomol GmbH, Hamburg, Germany
8R/S-HETE	Biomol GmbH, Hamburg, Germany
Free cholesterol	Serva Electrophoresis GmbH, Heidelberg, Germany
Cholesterol acetate	Santa Cruz Biotechnology, Dallas, USA
Cholesterol stearate	Merck, Taufkirchen, Germany
Cholesterol oleate	Merck, Taufkirchen, Germany
Cholesterol linoleate	Merck, Taufkirchen, Germany
Cholesterol linolenate	MedChemExpress, Monmouth Junction, USA
Cholesterol arachidonate	Merck, Taufkirchen, Germany
DPBS	PAN-Biotech, Aidenbach, Germany
Oil red O	Merck KGaA, Taufkirchen, Germany
Sodium borohydride	Life Technologies, Inc., Eggenstein, Germany
Isopropyl-β-thiogalactopyranoside (IPTG)	Carl Roth GmbH, Karlsruhe, Germany
Restriction endonucleases	ThermoFisher, Schwerte, Germany
E. coli strain Rosetta2 DE3 pLysS	Novagen, Darmstadt, Germany
DNA oligonucleotides (primers)	Eurofins MWG Operon, Ebersberg, Germany
Enpresso® B kit,	Enpresso GmbH, Berlin, Germany
dNTPs Mix	Bioline, Meridian Bioscience, Memphis, TN, USA
Oligo(dT)$_{18}$ Primer	BioTeZ Berlin-Buch GmbH, Berlin, Germany
RNAlater® Stabilization Solution	Sigma-Aldrich, Merck KGaA, Taufkirchen, Germany
TRI Reagent Solution	Invitrogen, Thermo Fisher, Waltham, MA, USA
Isoflurane	CP-Pharma, Burgdorf, Germany
GelRed® Nucleic Acid Gel Stain	Biotium, Fremont, CA, USA
5x HOT FIREPol® Blend Master Mix	Solis BioDyne, Tartu, Estonia
PCR Clean-up Kit	Macherey-Nagel, Düren, Germany
RNase Inhibitor	Bioline, Meridian Bioscience, Memphis, TN, USA
Tetro Reverse Transcriptase	Bioline, Meridian Bioscience, Memphis, TN, USA

RNeasy Mini Kit	Qiagen GmbH, Hilden, Germany
SensiFAST SYBR® No-ROX Kit	Meridian Bioscience, Memphis, TN, USA

1.2. Mouse models used in this study (**Table S2**)

Strain	Vendor or Source	Background Strain	Sex	Other Information
ApoE$^{-/-}$	in house	C57BL/6J	M&F	
Alox15$^{ki/ki}$	in house	C57BL/6J	M&F	
Alox15$^{ki/ki}$ + *ApoE*$^{-/-}$	in house	C57BL/6J	M&F	Breeding scheme explained in text.

1.3. ARRIVE guidelines (**Table S3**)

Groups	Sex	Age	Number (prior to experiment)	Number (after termination)	Littermates (Yes/No)	Other description
**Group 1** (control, Alox15$^{wt/wt}$+ApoE$^{-/-}$)	F&M	14 and 25 weeks	15 per time point and sex	0	no	In house preparation
**Group 2** (experimental, Alox15$^{ki/ki}$+ApoE$^{-/-}$)	F&M	14 and 25 weeks	15 per time point and sex	0	no	In house preparation

Sample size: Prior sample size calculations were not possible since this study is exploratory and there is no previous data on the impact of humanization of mouse Alox15 on atherogenesis.

Inclusion Criteria: Good health status, average body weight, normal moving behavior, normal fur

Exclusion Criteria: obvious developmental irregularities, abnormal moving behavior, abnormal aggressiveness

Randomization: yes; within each genotype and sex, animals were randomly assigned to the 14-week or 25-week sampling cohort.

Blinding: no blinding

2. Methodological supplement

2.1. Preparation of the recombinant ALOX15 orthologs

Mouse Alox15 variants were expressed as N-terminal hexa-his-tag fusion proteins. For this, competent *E. coli* cells (strain Rosetta 2 DE3 pLysS) were transformed with 50-100 ng of the recombinant expression plasmids and the cells were grown overnight on kanamycin/chloramphenicol containing agar plates. Isolated bacterial clones were picked and two 1 mL bacterial liquid cultures (LB medium with 50 µg/mL kanamycin and 35 µg/mL chloramphenicol) were grown for 6-8 h at 37°C. An appropriate volume of the pre-cultures was then added to 50 mL of sterile culture medium (Enpresso® B kit, Enpresso GmbH, Berlin, Germany) in ultra-yield culture flasks (Thomson Instrument Company, Oceanside, USA) containing kanamycin (50 µg/mL) and chloramphenicol (35 µg/mL) to achieve an

OD_{600} of 0.1-0.15. The cells were grown overnight at 30°C and 250 rpm. After the OD_{600} had reached values above 5, expression of the recombinant proteins was induced by the addition of 1 mM (final concentration) IPTG and booster tablets as well as reagent A according to the instructions of the vendor (Enpresso® B kit). Then the cultures were maintained at 22°C for 18 h at 230-250 rpm agitation. Bacteria were harvested by centrifugation, the resulting pellet was reconstituted in a total volume of 5 mL PBS and bacteria were lysed by sonication using a Branson W-250P tip sonifyer (Heineman, Schwäbisch-Gmünd, Germany). Cell debris was spun down (15 min, 15,000 x g, 4°C), the supernatants were recovered, aliquoted and snap-frozen in liquid nitrogen. The recombinant his-tag fusion proteins were subsequently enriched by affinity chromatography on a Ni-agarose column and the final enzyme preparations were dialyzed against DPBS overnight at 4 °C to remove the imidazole that was used to remove the recombinant protein from the stationary phase of the affinity chromatography

2.2. In vitro ALOX15 activity assays

To assay the catalytic activity of the recombinant enzyme preparations, aliquots of the bacterial lysate supernatants or the dialyzed column eluate of the affinity chromatography were incubated in 0.5 mL DPBS containing 100 µM AA as substrate. After a 3 min incubation period the reaction was terminated by the addition of 1 mg solid sodium borohydride ($NaBH_4$), which inactivated the enzyme and simultaneously reduced the hydroperoxy fatty acids formed during the ALOX reaction to the more stable alcohols. After 5 min on ice 75 µL acetic acid and 0.5 mL of acetonitrile were added and denatured proteins were spun down. The aliquots of the protein-free supernatant were then injected to RP-HPLC for quantification of the AA oxygenation products.

RP-HPLC was carried out on a Shimadzu HPLC system equipped with a SIL-20AC auto-injector. The analytes were separated isocratically on an EC 250/4 Nucleodur 100-5 C18 column (Macherey-Nagel, Düren, Germany) connected with an EC 4/3 Nucleodur 100-5 C18 ec pre-column (Macherey-Nagel, Düren, Germany) at room temperature using a solvent flow rate of 1 mL/min. A mobile phase consisting of acetonitrile : water : acetic acid (70 :30 : 0.1) was used and the UV-absorbance at 235 nm was recorded. To convert the peak areas of the ALOX15 products formed during the 3 min incubation period into nmoles of ALOX15 products the intensity scale of the chromatographic system was calibrated by injecting known amounts of 15-HETE and a 5-point calibration curve was established. Since the molar extinction coefficient of all HETE isomers was similar this calibration curve was used to quantify all HETE isomers.

2.3. In vitro lipoprotein oxygenase activity assays

To compare the LDL and HDL oxygenase activities of recombinant ALOX15 variants corresponding in vitro activity assays were carried out. For this purpose, purified human LDL (0.5 mg LDL protein) was dissolved in 0.5 mL of DPBS in a 2 mL Eppendorf tube. The reaction was started by the addition of aliquots of the enzyme preparations exhibiting identical AA oxygenase activities. After a 15 min incubation period at room temperature, the ALOX reaction was terminated by the addition of 1 mg solid sodium borohydride. 35 µL of glacial acetic acid were added and the LDL lipids were extracted. For this purpose, the samples were transferred to 20 mL glass tubes and 0.5 mL of DPBS was added. After mixing, 2.5 mL of methanol and 1.25 mL of chloroform were added and the sample was vortexed

for 1 min. At this time there was no phase separation. Then 1.25 mL of chloroform and 1.25 mL of DPBS were added and the samples were vortexed again for 1 min. To obtain good phase separation the samples were centrifuged at 5,000 g for 10 min were and the lower phase containing the total ester lipids was recovered. After evaporation of the solvents the remaining lipids were reconstituted in 100 µL 2-propanol. Finally, 300 µL of acetonitrile were added and this solution was stored at -20 °C under argon atmosphere for RP-HPLC based analysis of the cholesterol lipidomes.

For quantification of the oxygenated cholesterol esters aliquots of the total lipid extracts were injected into RP-HPLC with UV-detection. Separation of the different oxidized and non-oxidized cholesterol derivatives (free chol, chol-AA, chol-LeA, chol-LA, chol-OA, chol-SA) was carried out on a Nucleodur C18 ec column (250 × 4 mm, 5 µm; Macherey-Nagel GmbH & Co. KG, Düren, Germany) equipped with a guard column (8 × 4 mm, 5 µm; Macherey-Nagel GmbH & Co. KG, Düren, Germany). An isocratic elution system consisting of 65% acetonitrile and 35% 2-propanol (0.1 % acetic acid) was used. The flow rate was set to 1 mL/min and the column temperature was maintained at 45 °C. The amounts of cholesterol esters carrying an oxygenated PUFA residue (conjugated diene) were quantified as readout parameter for the LDL oxygenase activity of the two (human and mouse ALOX15) enzyme preparations.

2.4. Preparation of peritoneal lavage cells and ex vivo Alox15 activity assays

To prepare peritoneal lavage cells 10 mL of DPBS were injected into the peritoneal cavity of sacrificed Alox15$^{ki/ki}$ + ApoE$^{-/-}$ mice and outbred Alox15$^{wt/wt}$ + ApoE$^{-/-}$ control animals. The abdomen was gently massaged and the lavage fluid was subsequently aspirated. On average, 7-8 mL of lavage fluid was recovered. The cell suspension was centrifuged at 800 × g for 10 min at room temperature, the supernatant was discarded and the cell pellet was resuspended in 5 mL of DPBS. This washing procedure was repeated three times. After the final wash, the cells were reconstituted in 1 mL of DPBS, counted and aliquots of the cell suspension were used for cellular *ex vivo* Alox15 activity assays.

For this purpose, an aliquot of the cell suspension involving 1-2 x 10^6 cells was incubated in 0.5 mL of DPBS containing 100 µM AA as substrate. This mixture was incubated at room temperature for 15 min. Hydroperoxy AA derivatives formed during the incubation period were reduced to the more stable hydroxy compounds by the addition of 1 mg of solid sodium borohydride ($NaBH_4$). The mixture was incubated for 5 min on ice and then 35 µL of glacial acetic acid and 500 µl of acetonitrile were added. The sample was vortexed and denatured proteins were removed by centrifugation (10 min at 4 °C at 18,000 g).

For quantification of the Alox15 products formed during the incubation period the protein-free supernatants were analyzed by RP-HPLC, which was performed on a Shimadzu HPLC system equipped with a Nucleodur C18 Gravity column (250 × 4 mm, 5 µm; Macherey-Nagel, Germany). As mobile phase (isocratic elution) a solvent mixture consisting of 70% acetonitrile, 30% water, 0.1% acetic acid was used at a flow rate of 1 mL/min at 25 °C. Compounds carrying conjugated dienes eluted in the retention time range of 7 - 10 min were collected, the solvents were evaporated and the analytes were reconstituted in 200 µL of the solvent system used for combined normal phase / chiral phase HPLC (NP/CP-HPLC). For analysis of the enantiomer composition of the major AA oxygenation products combined NP/CP-HPLC was carried out on a Chiralpak AD-H column (4.6 × 250 mm, 5 µm; Daicel,

Japan) with a mobile phase (isocratic elution) consisting of a mixture of n-hexane : methanol : ethanol : acetic acid (96 : 3 : 1 :0.1, by vol). For preparation of the mobile phase methanol, ethanol and acetic acid were mixed and this mixture was added to n-hexane.

2.5. Extraction of total lipids from blood plasma and from aortic tissue

100 µL of blood plasma were diluted with 0.5 mL of DPBS and the samples were supplemented with 100 µl of an ethanolic solution (0.4 mg/mL) of cholesteryl acetate serving as external extraction standard. Then 1.5 mL of methanol and 0.75 mL of chloroform were added, the mixtures were transferred to 5 mL Eppendorf tubes and vortexed for 2 min in 4 consecutive 30 s cycles. Under these conditions (single-phase lipid extraction) no phase separation was observed. After 30 min on ice 0.75 mL of chloroform and 0.75 mL of DPBS were added, the samples were vortexed again for 3 times for 30 s min and complete phase separation was achieved by centrifugation (5 min at 5,000 g). The lower chloroform phase was recovered, the solvents were evaporated and the remaining lipids were reconstituted in 100 µL 2-propanol. Finally, 300 µL of acetonitrile were added and this solution was stored at -20 °C under argon atmosphere for RP-HPLC based analysis of the cholesterol lipidomes.

For effective extraction of the total tissue lipids the aortic segments were added to 1.5 mL of methanol and the samples were supplemented with 100 µL of the external extraction standard solution. This mixture was then homogenized in Lysing Matrix D tube using the FastPrep-24 homogenizer. Three homogenization cycles at 6.5 m/s for 30 s were run with 5-minute cooling intervals on ice in between the cycles. The tissue homogenates were subsequently transferred to 5 mL Eppendorf tubes, mixed with 0.6 mL DPBS and 0.75 mL chloroform and vortexed thoroughly 4 times for 30 s with intermediate cooling on ice. During this first extraction step, no phase separation was observed (single phase lipid extraction). After 30 min cooling period on ice 0.75 mL of chloroform and 0.75 mL of DPBS were added, the samples were vortexed again for 3 times for 30 s min and complete phase separation was achieved by centrifugation (5 min at 5,000 g). The lower chloroform phase was recovered, the solvents were evaporated and the remaining lipids were reconstituted in 100 µL 2-propanol. Finally, 300 µL of acetonitrile were added and this solution was stored at -20 °C under argon atmosphere for RP-HPLC based analysis of the cholesterol lipidomes.

2.6. Genotyping

Genotyping of the *Alox15* and the *ApoE* gene loci was carried out by genomic PCR and the following gene-specific amplification and sequencing primers were used. For genotyping of the ApoE locus the upstream primer was identical for both, the wildtype allele and the knockout allele. The downstream primer was different for the two genotypes and thus, the amplification products of the wild-type and the knock-out alleles were of different size. For genotyping of the Alox15 gene locus, the region of the locus carrying the mutation was amplified by genomic PCR and the amplification product was isolated by agarose gel electrophoresis and nucleotide sequence was determined. For genomic PCR the extracted DNA samples were first heated to 95 °C for 15 min and then 29 amplification cycles were run each consisting of 20 s denaturation phase (95 °C), 30 s annealing phase (65 °C) and a 60 s synthesis phase (72 °C). After the last amplification cycle the sample temperature was kept for 10 min at 72 °C and then the samples were cooled down to 10 °C for storage.

Table S4: Sequences of the genotyping primers used for amplification and sequencing of the Alox15 and the ApoE gene loci.

Purpose	Location	Sequence
Alox15 genotyping	upstream	TAG GCA CCC AGG TAG GCT TTG GT
	downstream	CTG AAC CCT GCC TGA GAT GCC AC
Alox15 sequencing		GAG GAA GTA GGG ATC GCT GAG CC
ApoE wildtype	upstream	GCC TAG CCG AGG GAG AGC CG
	downstream	TGT GAC TTG GGA GCT CTG CAG C
ApoE knockout	upstream	GCC TAG CCG AGG GAG AGC CG
	downstream	GCC GCC CCG ACT GCA TCT

2.7. Total RNA extraction and qRT-PCR

To obtain high-quality RNA preparations, the aortic arch tissue was preserved in RNAlater immediately after removal from the sacrificed mice. After thawing, the tissue was chopped into small pieces with a pair of scissors, transferred to Matrix D tubes, and homogenized in 700 µL of TRIzol solution using a FastPrep-24 homogenizer (6.5 m/s for 3 cycles of 30 s each, with 5-minute intervals on ice between cycles). The tissue homogenate was transferred to a new 1.5 mL Eppendorf tube, 140 µl of chloroform were added and the samples were vortexed rigorously. Then the homogenate was incubated at room temperature for 3 min and centrifuged at 12,000 g for 15 min at 4 °C. The upper aqueous phase was transferred to a new 1.5 mL Eppendorf tube and an equal volume of 70% ethanol was added. Subsequently the RNA was purified following the manufacturer's protocol of the RNeasy Mini Kit (Qiagen GmbH, Hilden, Germany). Finally, the RNA was eluted in 30 µL of RNase-free water. The RNA concentrations and the degree of purity were determined spectrophotometrically using the A_{260}/A_{280} method and the RNA extracts were stored at -80 °C. For reverse transcription aliquots of the RNA preparation were mixed with water to adjust a final concentration of 40 ng/µL. 12.5 µL of this dilution was then combined with 7.5 µL of the reverse transcription Mix (Bioline, Meridian Bioscience, Memphis, TN, USA). The mixture was incubated at 45 °C for 60 min, followed by an incubation at 85 °C for 5 min to terminate the reaction. Subsequently, the sample was cooled to 10 °C and the resulting cDNA was stored at -20 °C.

For qRT-PCR of the pro- and anti-inflammatory gene products pairs of specific amplification primers were designed and their nucleotide sequence is given in the Methodological supplement. For qRT-PCR, 1 µl of the cDNA preparation, 5 µL of the 2-fold concentrated SensiFAST SYBR No-ROX Mix (Meridian Bioscience, Memphis, USA) and 4 µL of water were added to the PCR tubes and the

amplification conditions were as follows. The initial denaturation phase (10 min at 95 °C) was followed by 40 amplification cycles each consisting of a 15 s denaturation phase (95 °C), a 30 s annealing phase (65 °C) and a 20 s synthesis phase (72 °C). Finally, the samples were kept for 1 min at 60 °C and then cooled down to 10 °C. Melting curves were continuously recorded. The sequences of the gene-specific primer combinations used for amplification of the reference and target genes are specified in **Table S5**.

Table S5: Gene-specific primer combinations used for amplification of the steady-state mRNA concentrations.

Gene	Location	Sequence
Hprt (hypoxanthin-phosphoribosyl-transferase 1, reference gene)	upstream	CCC AGC GTC GTG ATT AGC GAT GA
	downstream	GAG CAA GTC TTT CAG TCC TGT CCA
iNOS (inducible nitric oxide synthase)	upstream	TGA GGC CCA GGA GGA GAG AGA
	downstream	TCA CAG GCT GCC CGG AAG GTT
PTGS2 (prostaglandin synthase 2)	upstream	GCA TTC TTT GCC CAG CAC TTC AC
	downstream	CCA AAG ACC TCC TGC CCC ACA
Tnf_{alpha} (tumor necrosis factor alpha)	upstream	CAC CAT GAG CAC TGA AAG CAT GAT
	downstream	CGG CTG ATG GTG TGG GTG AGG
Tgf_{beta} (transforming growth factor $_{beta}$)	upstream	CGG CCC TTC CTG CTC CTC ATG G
	downstream	CAG GAG CGC ACG ATC ATG TTG GA
IL10 (interleukin 10)	upstream	GCC AGG TGA AGA CTT TCT TTC AAA CA
	downstream	GCT TCT CAC CCA GGG AAT TCA AAT GC
Arg 1 (arginase 1)	upstream	CGC CTT TCT CAA AAG GAC AGC CTC
	downstream	CAC ACC AGC CAG CTC TTC ATT GG

3. Supplemental experimental data

3.1. Calibration parameters of the cholesterol lipidome analytical system

The cholesterol lipidomes of the blood plasma and of the aortic tissue were quantified by an RP-HPLC-based method using UV-detection. The concentrations of the following metabolites were measured: i) free cholesterol, ii) Cholesterol acetate (cholesterol ester of acetic acid; this compound does not occur in biological samples but was added to the samples to quantify the extraction efficiency

of the cholesterol derivatives). iii) Cholesteryl linolenate (cholesterol ester of linolenic acid, C18:Δ3,n-3), iv) Cholesteryl arachidonate (cholesterol ester of arachidonic acid, C20: Δ 4,n-6), v) Cholesteryl linoleate (cholesterol ester of linoleic acid, C18: Δ 2,n-6), vi) Cholesteryl oleate (cholesterol ester of oleic acid, C18: Δ 1,n-9), vii) Cholesteryl stearate (cholesterol ester of stearic acid, C18: Δ0). To calibrate our RP-HPLC system known amounts of the different compounds were injected to RP-HPLC and the retention times (min) and the peak areas were quantified. The different measuring points were evaluated by linear regression analysis and the correlation coefficients (R^2) were determined. The different molar extinction coefficients of the different cholesterol derivatives depend on the number of double bonds present in the compounds.

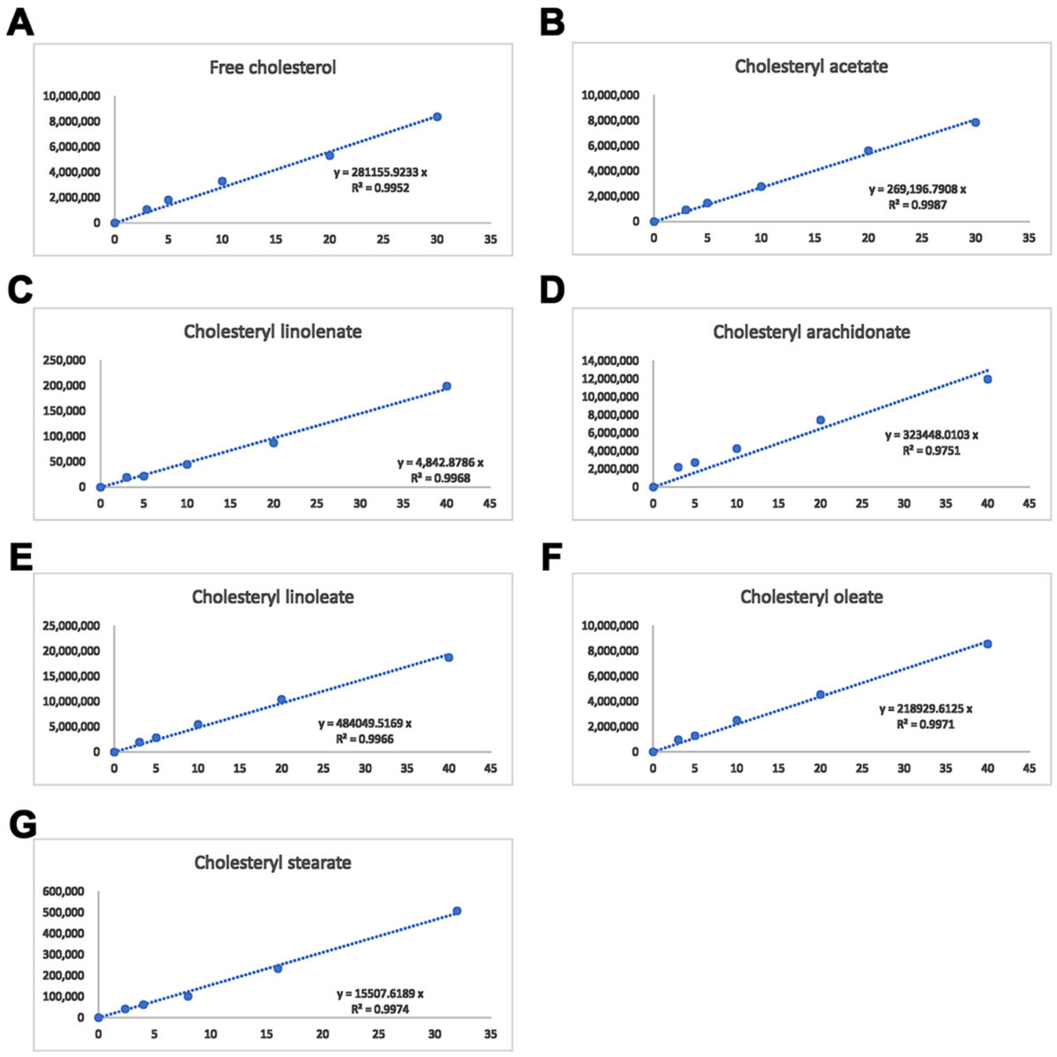

Figure S1. Calibration curves of the different cholesterol derivatives. Known amounts of the different cholesterol derivatives were injected into RP-HPLC and 6-point calibration curves for each compound were recorded. The different measuring points were evaluated by linear regression analysis and the correlation coefficients R^2 were evaluated. These 6-point calibration curves were then used to convert the peak areas determined by RP-HPLC for the different biological samples (blood plasma, aortic tissues) into nmoles cholesterol derivative per mL blood plasma (quantification of the degree of hypercholesterolemia) and into nmoles of cholesterol derivative per mg aortic tissue wet weight (quantification of the degree of lipid deposition in the aortic wall). For these calculations, the formulas

and the molecular weights of the different cholesterol derivatives are required and the numeric values for these parameters are given in **Table S6**.

Table S6. Calibration parameter of the cholesterol lipidome system. The area units of our cholesterol lipidome analyses were converted into nmoles cholesterol derivative using the following parameters.

Compounds	Formular	Molecular weight (g/mol)	HPLC area unit/µg	HPLC area unit/nmol
Free cholesterol	$C_{27}H_{46}O$	386.66	281,156	108,712
Cholesteryl acetate	$C_{29}H_{48}O_2$	428.69	269,197	115,402
Cholesteryl linolenate	$C_{45}H_{74}O_2$	647.07	288169	186,466
Cholesteryl arachidonate	$C_{47}H_{76}O_2$	675.12	323,448	218,366
Cholesteryl linoleate	$C_{45}H_{76}O_2$	649.08	484,050	314,187
Cholesteryl oleate	$C_{45}H_{78}O_2$	651.12	218,930	142,549
Cholesteryl stearate	$C_{45}H_{80}O_2$	653.12	15,508	10,128

From the 6-point calibration curves **(Figure S1**) the peak areas determined for the biological samples were converted into nmoles of cholesterol derivative per mL blood plasma or to nmoles cholesterol derivative per mg wet weight of aortic tissue. These readout parameters were then employed to quantify the degree of hypercholesterolemia (blood plasma) of the individual mice and the degree of lipid deposition in the different sections of the aortic tissue.

3.2. Normalization of the recombinant ALOX preparations

For the in vitro lipoprotein oxygenase activity assays, we prepared recombinant human ALOX15, recombinant mouse Alox15 and the functionally humanized Leu353Phe mutant of mouse Alox15 in E. coli and used the bacterial lysate supernatant as enzyme source. However, since the expression efficiencies of the three enzymes were different, we first determined the ALOX concentration in the bacterial lysate supernatants. For this purpose, we applied 5 µL of the bacterial lysates to SDS-PAGE and probed the protein blots using an anti-his-tag antibody that specifically stains the ALOX bands. To calibrate the band intensity scale, we also applied 1 µg of recombinant ALOX from *M. fulvus* to SDS-PAGE. This enzyme was also expressed as N-terminal hexa-his-tag fusion protein and has subsequently been purified to electrophoretic homogeneity by affinity chromatography on an open-bed Ni-agarose column **(Figure S2)**. Using this calibration procedure, we determined the amounts of ALOX

proteins in the lysate supernatants of the three enzyme preparations and the numeric values are given below the immunoblot in **Figure S2**. For our *in vitro* lipoprotein oxygenation experiments (**Figure 1** of the main paper) we used identical amounts of ALOX protein of the different enzyme preparations.

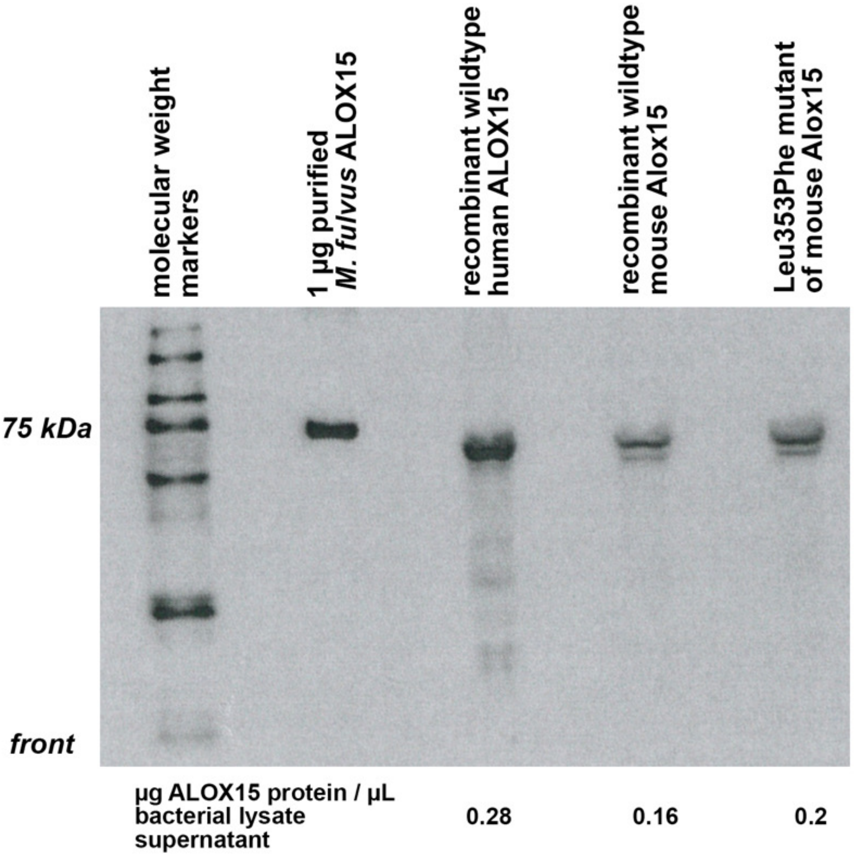

Figure S2. Immunoblotting of the recombinant ALOX15 preparations. To quantify the ALOX15 concentrations in the bacterial lysates we analyzed the lysate by quantitative immunoblotting using the recombinant ALOX-isoform of *Myxococcus fulvus* (M. fulvus) as calibration standard. The intensities of the ALOX15 bands of the Western blot were quantified and the ALOX concentrations in the bacterial lysate supernatants were calculated. The numeric values are given below the immunoblot. For the in vitro lipoprotein oxygenase activity assays different volumes of the lysate supernatants were employed so that the ALOX15 concentration in the activity assays was identical.

3.3. HDL oxygenase activity of mouse Alox15 variants

Aliquots of recombinant Alox15 preparations (bacterial lysate supernatants normalized to the same amounts of recombinant his-tag fusion protein) were incubated for 15 min in 0.5 mL DPBS containing 1 mg/mL of HDL protein. After the incubation period the peroxy products formed during the incubation period were reduced, the samples were acidified, the total lipids were extracted and the extracts were hydrolyzed under alkaline conditions. The resulting fatty acid derivatives were analyzed by RP-HPLC, which was carried out as described for the products of the LDL oxygenase assays (see chapter 2.3 of this Supplement). Each chromatogram was followed at 235 nm to detect the ALOX15 products (13-HODE, 15-HETE, 12-HETE) and at 210 nm to quantify the amounts of unoxidized PUFAs.

From this data we calculated the OH-PUFA / PUFA ratio, which is a suitable measure for the oxidation degree of the HDL lipids **(Figure 1G)**.

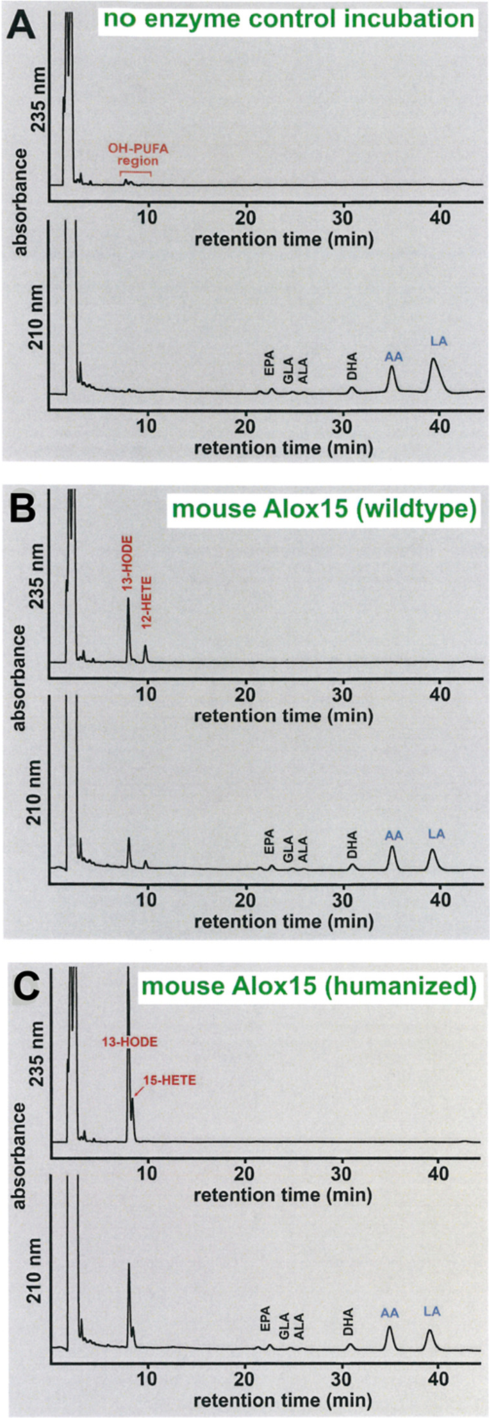

Figure S3. In vitro HDL oxygenase activities of ALOX15 variants. *In vitro* lipoprotein oxygenase activity assays were carried out as described in the Methodological Supplement. Enzyme preparations were normalized for an equal ALOX15 protein content of the different enzyme preparations. Two independent activity assays (n=2) were run for each enzyme preparation and representative RP-HPLC chromatograms are shown. **A)** No enzyme control incubation. **B**) Recombinant wild-type mouse Alox15 was used as catalyst. **C)** Recombinant Leu353Phe mutant of mouse Alox15 (functionally humanized

mouse Alox15) was used as catalyst. EPA, eicosapentaenoic acid; GLA, gamma linolenic acid; ALA alpha linoleic acid; DHA, docosahexaenoic acid; AA, arachidonic acid; LA, linoleic acid.

3.4. Analysis of the enantiomer composition of the major oxygenation products formed by humanized mouse Alox15 from human lipoproteins (LDL, HDL).

Auto-oxidation of PUFAs leads to a complex mixture of oxygenation products and the different positional isomers are racemic mixtures, in which the two enantiomers (S- and R-enantiomers) are present in similar amounts. In contrast, ALOX-isoforms oxygenate their substrates to chiral oxygenation products and for most mammalian ALOX-isoforms (except for ALOX12B) the S-enantiomer is dominant. To test the enantiomer composition of the major oxygenation products formed from LDL and HDL by the humanized mouse Alox15 (Leu353Phe mutant of mouse Alox15) we incubated the recombinant enzyme (bacterial lysate supernatant) for 15 min in the presence of 1 mg/mL lipoproteins (LDL, HDL), prepared the major oxygenation products by RP-HPLC and analyzed them by combined normal-phase / chiral phase HPLC (NP/CP-HPLC. Here we found **(Figure S3)** that the two major oxygenation products (13-HODE, 15-HETE) were chiral since 13*S*-HODE and 15*S*-HETE were formed in large amounts. In contrast, only small amounts of the corresponding R-enantiomers (13*R*-HODE, 15*R*-HETE) were detected. This data confirms the enzymatic origin of the lipoprotein oxygenation products and excludes auto-oxidation as metabolic route.

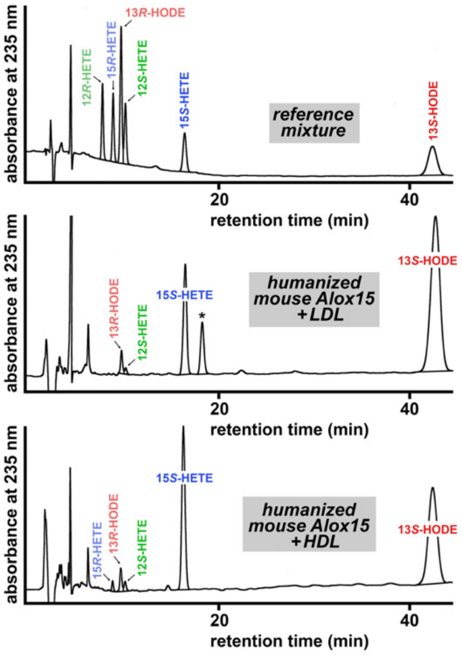

Figure S4. Enantiomer composition of the lipoprotein oxygenation products formed *in vitro* from human LDL and HDL by the functionally humanized Leu353Phe mutant of mouse Alox15. *In vitro* lipoprotein oxygenase activity assays were carried out as described in the Methodological Supplement. Two independent activity assays (n=2) were run for each lipoprotein preparation. The oxygenation products (conjugated dienes) were prepared by RP-HPLC chromatography and further analyzed by combined NP/CP-HPLC. Upper trace: Racemic reference compounds. Here, similar amounts of S- and R-enantiomers of 12*S/R*-HETE, 15*R/S*-HETE and 13*R/S*-HODE were analyzed. Middle trace: Product composition formed from human LDL by humanized mouse Alox15. 13*S*-HODE and 15*S*-HETE were

the major oxygenation products and the corresponding *R*-enantiomers were only present in small amounts. Lower trace: Product composition formed from human HDL by humanized mouse Alox15. Here again, 13*S*-HODE and 15*S*-HETE were the major oxygenation products and the corresponding *R*-enantiomers were only present in small amounts. * unknown compound (no conjugated diene).

3.5. Alox15$^{ki/ki}$ + ApoE$^{-/-}$ mice do not show morphological abnormalities

Alox15$^{ki/ki}$ + ApoE$^{-/-}$ mice express the functionally humanized Leu353Phe mutant of mouse Alox15 instead of the wildtype mouse Alox15 and this enzyme mutant exhibits the same functional properties as human ALOX15. When crossed into an ApoE$^{-/-}$ background the Alox15$^{ki/ki}$ + ApoE$^{-/-}$ mice are viable and do not show functional and structural abnormalities.

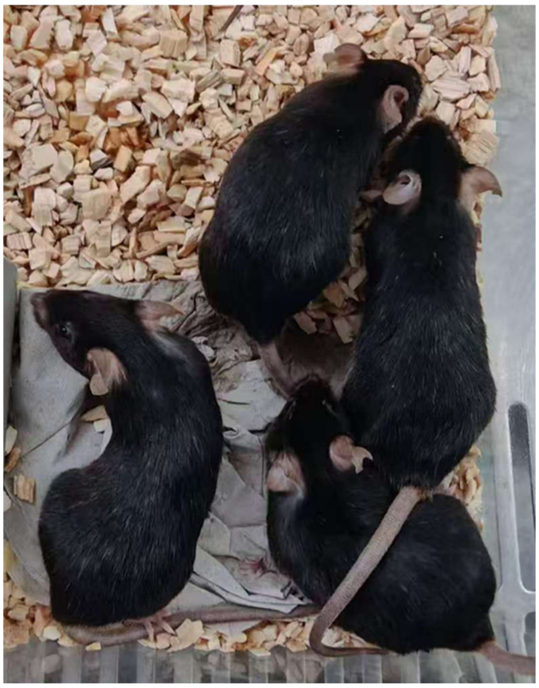

Figure S5. Alox15$^{ki/ki}$ + ApoE$^{-/-}$ mice. Representative individuals of our Alox15$^{ki/ki}$ + ApoE$^{-/-}$ colony were randomly selected and a picture was taken.
